# Micro–Nano Hierarchical Structure Enhanced Strong Wet Friction Surface Inspired by Tree Frogs

**DOI:** 10.1002/advs.202001125

**Published:** 2020-08-09

**Authors:** Liwen Zhang, Huawei Chen, Yurun Guo, Yan Wang, Yonggang Jiang, Deyuan Zhang, Liran Ma, Jianbin Luo, Lei Jiang

**Affiliations:** ^1^ School of Mechanical Engineering and Automation Beihang University Beijing 100191 China; ^2^ Beijing Advanced Innovation Center for Biomedical Engineering Beihang University Beijing 100191 China; ^3^ State Key Laboratory of Tribology Tsinghua University Beijing 100091 China; ^4^ Laboratory of Bioinspired Smart Interface Science Technical Institute of Physics and Chemistry Chinese Academy of Sciences Beijing 100190 China

**Keywords:** bio‐interfaces, interfacial liquid adjustment, tree frogs, wearable sensors, wet friction

## Abstract

Superior wet attachment and friction performance without the need of special external or preloaded normal force, similar to the tree frog's toe pad, is highly essential for biomedical engineering, wearable flexible electronics, etc. Although various pillar surfaces are proposed to enhance wet adhesion or friction, their mechanisms remain on micropillar arrays to extrude interfacial liquid via an external force. Here, two‐level micropillar arrays with nanocavities on top are discovered on the toe pads of a tree frog, and they exhibit strong boundary friction ≈20 times higher than dry and wet friction without the need of a special external or preloaded normal force. Microscale in situ observations show that the specific micro–nano hierarchical pillars in turn trigger three‐level liquid adjusting phenomena, including two‐level liquid self‐splitting and liquid self‐sucking effects. Under these effects, uniform nanometer‐thick liquid bridges form spontaneously on all pillars to generate strong boundary friction, which can be ≈2 times higher than for single‐level pillar surfaces and ≈3.5 times higher than for smooth surfaces. Finally, theoretical models of boundary friction in terms of self‐splitting and self‐sucking are built to reveal the importance of liquid behavior induced by micro–nano hierarchical structure.

## Graphical abstract

A strong wet attachment bioinspired surface with hierarchical pillars and nanocavities is introduced based on the unique interfacial liquid adjusting effects on the tree frog toe pad, where robust interfacial capillarity from nanometer‐thick liquid film generates a boundary friction ≈20 times its wet and dry friction. Such bioinspired surfaces demonstrate potential applications in fields including medical devices and wearable sensors.

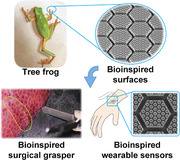

## 1 Introduction

Undesirable slipping is a common phenomenon occurring on the contact surfaces especially in the presence of liquid film. Such unexpected slipping behavior could result in severe consequences such as soft tissue damage in surgical grasping,^[^ ^1^, ^2^, ^3^, ^4^, ^5^ ^]^ or vehicle wheel spinning in traffic accidents.^[^ ^6^, ^7^, ^8^ ^]^ Particularly in the emerging field of wearable flexible electronics, the wet contact issues become more dominant due to the accumulation of sweat, mucus or even blood on the surface of biological tissue. In order to prevent undesirable slipping, superior wet friction and attachment performances are in great demand on wet surfaces and have attracted worldwide attention owing to their diverse potential applications in medical engineering, bioengineering, and wearable flexible electronics.^[^ ^9^, ^10^, ^11^, ^12^, ^13^, ^14^, ^15^ ^]^

Attempts to improve the surface performance of wet attachment and friction have mainly been pursued by the use of special adhesives or octopus‐inspired sucker structures.^[^ ^15^, ^16^, ^17^, ^18^, ^19^, ^20^ ^]^ To resolve the drawbacks that existing dry adhesives are unsuitable for wet surfaces, bioinspired two‐layer adhesives have been created combining dissipative materials and surface structures to achieve high adhesion energy on various wet and dynamic surfaces;^[^ ^16^, ^17^, ^18^ ^]^ however, the reuse of adhesives is a bottleneck for applications in wearable flexible electronics because accumulated contaminants from skin can weaken the adhesive chemical bonds or Van der Waals forces. As another typical method for wet adhesion, the octopus‐inspired sucker structure works through the generation of vacuum pressure; whereas a certain amount of external preloaded normal force, 20–50% of friction force, is necessary to extrude interfacial air or liquid.^[^ ^15^, ^19^, ^20^ ^]^ Any excessive external force should be avoided as much as possible due to the vulnerability of soft biological tissue. Therefore, it is highly desirable to develop a reusable adhesive surface with extraordinary wet friction and attachment functions, which can be generated automatically with little external force.

Fortunately, tree frogs with a strong wet attachment ability can freely and repeatedly climb on vertical surfaces or overhang without a special external or preloaded normal force.^[^ ^21^, ^22^ ^]^ Previous discussions of tree frog toe pads have focused on their hierarchical pillar structures forming an interfacial liquid drainage strategy, where an external normal force with strength of 20–100% of the friction force is always desired to extrude the interfacial liquid and create a firm solid attachment.^[^ ^23^, ^24^, ^25^ ^]^ Thus, the wet attachment mechanisms of tree frogs in nature may not be have been sufficiently revealed. In addition, the hierarchical micro–nano structures and their cooperative actions on interfacial liquid have been ignored. In this work, a significant increasing in wet friction (≈20 times) has been observed while the interfacial liquid volume was decreased on tree frog's toe pad without applying of a special external or preloaded normal force. This phenomenon indicates that the interfacial liquid in certain states is likely to enhance the wet friction substantially rather than lead to slipping. By revealing these interfacial liquid adjusting mechanisms on tree frog's toe pad, bioinspired surfaces could be developed with novel strong wet attaching strategies.

## 2 Results and Discussion

### 2.1 Liquid Adjusting Phenomena and Strong Boundary Friction on Tree Frog's Toe Pad

**Figure 1:**
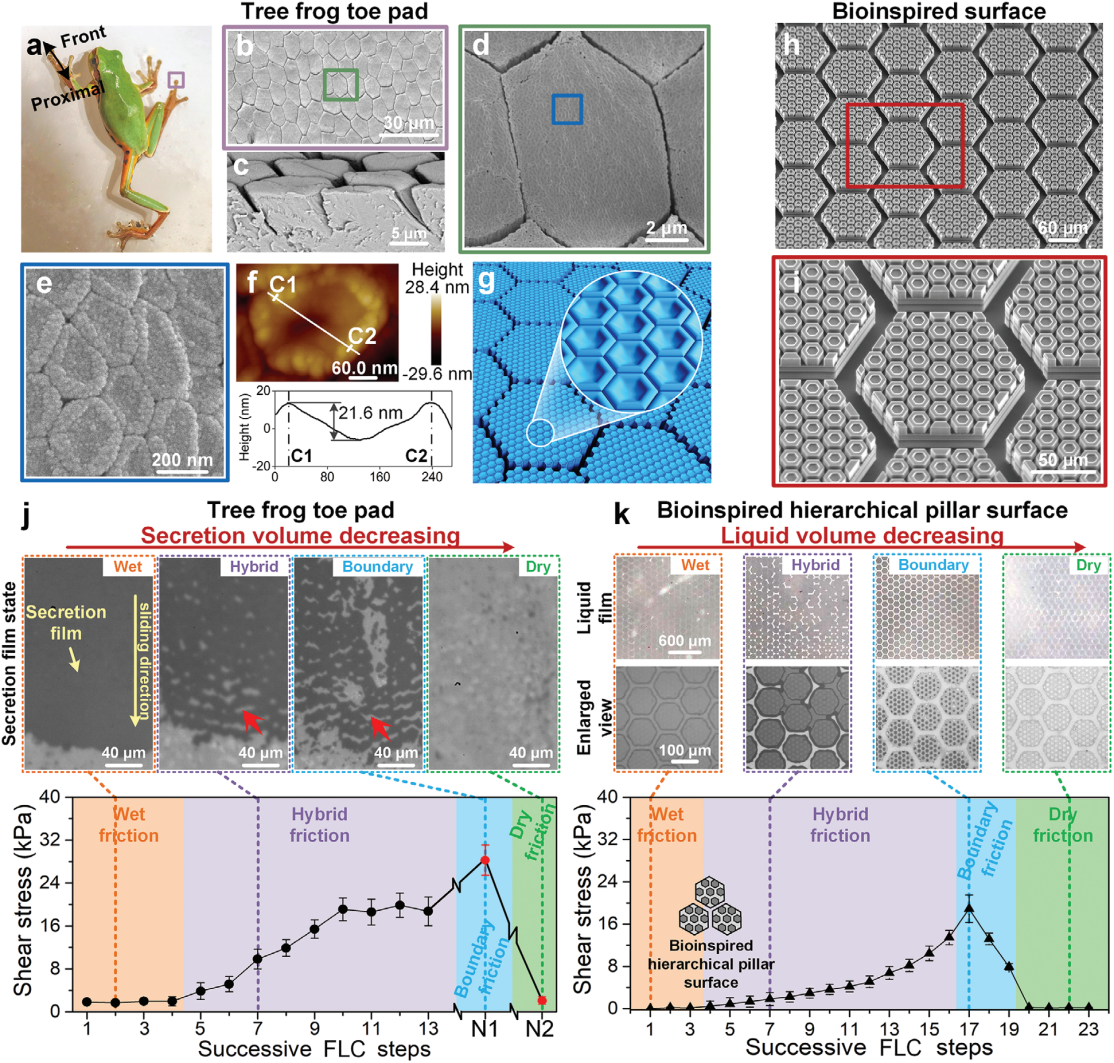
Structural characterization of the tree frog's toe pad. a) Tree frog, *Polypedates* and *Rhacophorus dennysi*, climbs on a wet smooth glass. b) The top pad is covered with micropillar epithelial cells, with a primarily hexagonal pattern with a diameter of ≈10 µm. c) Sectional view of toe pad shows the height of pillar is ≈5 µm. d,e) Nanopillars tightly arrange on each micropillar. f) The atomic force microscope image demonstrates that the top of the nanopillar is concave with a depth of 20 ± 7 nm (sample size *n* = 50). g) Typical feature structures of the toe pad. h,i) Artificial bioinspired hierarchical concave pillar surface with two‐level pillars and cavities (Figure S2 and Table S1, Supporting Information). j) During the FLC steps (Figure S3c, Supporting Information), wet, hybrid, boundary, and dry liquid film states appear in turn, corresponding to their wet, hybrid, boundary, and dry friction. The dark area is the secretion film between the toe pad and substrate. The red arrow denotes the secretion‐free channels. The friction in each step is measured with a normal load of ≈1 kPa and a toe pad contact area of ≈4 mm^2^. *N*_1_ and *N*_2_ indicate dabbing the toe pad on dust‐free papers 10 and 20 times, respectively. k) With 1.5 µL deionized water added, similar liquid film states and friction variation tendencies emerge on the hydrophilic bioinspired surface during FLC steps with a normal pressure of 100 Pa. Data are presented as mean ± SD (for (i) and (j), sample size *n* = 10).

Tree frogs (*Polypedates and Rhacophorus dennysi*) with strong wet attachment and friction can freely climb on vertical smooth surface for insect capturing or escaping without any external or preloaded normal force (**Figure** **1**a). As a typical natural biointerface, their toe pads surface has been extracted into micro–nano two‐level pillar arrays with nanocavities on top of each pillar (Figure 1g). The first‐level micropillars are primarily hexagon with diameter, height and channel width of about 10, 5, and 0.5 µm (Figure 1b,c).^[^ ^26^ ^]^ The second‐level hexagonal nanopillars have a diameter of ≈250 nm (Figure 1d,e), and nanoscale cavity with depth of ≈20 nm is on top (Figure 1f). Inspired by tree frog's structural features, various bioinspired pillar surfaces have been fabricated with polydimethylsiloxane (PDMS) to study the interfacial liquid behavior and its effects, including single‐level pillar surface, hierarchical pillar surface and hierarchical concave pillar surface (Figure 1h,i; Figures S1 and S2 and Table S1, Supporting Information). The diameter and height of first‐level pillars are 120 and 30 µm, and that of second‐level pillar are 20 and 10 µm, respectively. On hierarchical concave pillar surfaces, three kinds of cavity were prepared on second‐level pillar with the diameters of 6, 12, and 15 µm, and the depth of 3 µm.

To investigate the influence of interfacial liquid on wet attachment, successive friction tests were conducted on the toe pad of a living tree frog to mimic its crawling steps (Figure S3a–c and Movie S1, Supporting Information). The friction force and the states of the secretion film were simultaneously monitored under a small normal load of 5 mN during successive frog‐like‐crawling (FLC) steps, i.e., repetitive operations involving sliding a certain distance on a clean substrate and then lifting (Figure S3c, Supporting Information). With increasing FLC steps, the interfacial secretion volume declines regularly, and the secretion film state varies in turn from a continuous thick secretion film, a large‐patch discrete secretion film, a minor uniform discrete secretion film, to finally near dry, which hereafter are roughly defined as wet, hybrid, boundary and dry states (Figure 1j, upper; Figure S3d, Supporting Information). More interestingly, the friction is highly dependent on the interfacial secretion states and exhibits an obvious difference with the decline of secretion (Figure 1j, lower). In general, the friction increases and gradually reaches a peak as the secretion volume declines to the boundary state, then it significantly declines when the toe pad is wiped clean to near dry. The friction at the boundary state could be enhanced by ≈20 times higher than that of the wet or dry state, and its value could exceed ≈30 times its normal load (Figure S3e, Supporting Information). This wet contact friction phenomenon is completely different from usual friction decreasing or slipping induced by interfacial liquid. To eliminate the influence of uncontrollable secretion of tree frog toe pad (Figure S4, Supporting Information), various bioinspired surfaces were performed the FLC tests with deionized water. On bioinspired pillar surface with hydrophilicity, both the interfacial liquid states and the wet friction exhibit similar behaviors as the tree frog toe pads (Figure 1k; Figures S3f and S5b, Supporting Information), where the boundary friction is even enhanced up to ≈100 times higher than wet or dry friction and ≈200 times of its normal load. As compared to the tree frog, it becomes clear that liquid is distributed separately on top of the pillars to form uniform thin films in the boundary state, and this separated distribution has a deep relationship with the strong boundary friction. Therefore, it is necessary to reveal the underlying mechanism of the liquid boundary state at the microscale.

### 2.2 Characterization and Formation of Two‐Level Liquid Self‐Splitting Effect

**Figure 2:**
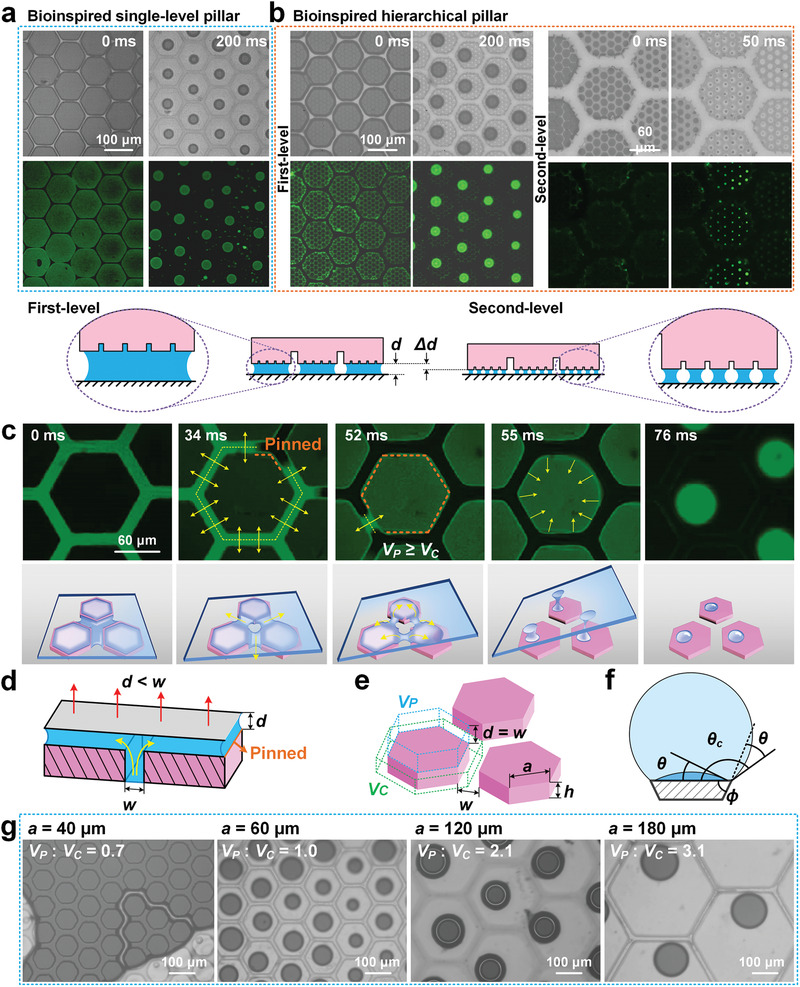
Characteristics of liquid movement and liquid self‐splitting on pillar surface during FLC steps. a) Liquid self‐splitting appears on bioinspired single‐level pillar surface during successive FLC steps. b) On bioinspired hierarchical pillar surface, two‐level self‐splitting in turn appears on first‐ and second‐level pillars with liquid volume decreasing, accompanied with gap distance *d* between pillar top and substrate decreases to *d* − Δ*d*. After self‐splitting, uniform liquid bridges forms on each pillar. c) During pillar lifting from substrate, liquid is continuously extracted from channels to pillar top, then separated into liquid bridge and forms self‐splitting effect. Yellow arrows denote the flowing direction of liquid film. d) Liquid first flows from channels to the gap between pillar top and substrate since gap distance *d* is smaller than channel width *w*. e) *V*_C_ is the liquid volume in channels around a pillar at *d* = 0, and *V*_P_ is the liquid volume on each pillar when *d* reaches to *w*. *a* is the side length of hexagonal pillar. *h* is pillar height. f) The critical contact angle *θ*_c_ can be presented as 90° + *θ + ϕ*, where *θ* is the natural contact angle of surface and *ϕ* is the angle of edge. With the edge pinning effect, liquid on pillar top is prevented to flow back to channels. g) Different bioinspired pillar surfaces are designed with *w* of 20 µm, *h* of 30 µm, and varied *a* from 40 to 180 µm. A continuous liquid film forms with *V*_P_ < *V*_C_, while self‐splitting liquid films appear with *V*_P_ ≥ *V*_C_.

Microscope and fluoroscope observations were conducted to observe the interfacial liquid motion in real time during successive FLC steps. Through the process of continuous steps, the interfacial liquid gradually declines and begins to distribute separately on pillars’ top. On the bioinspired single‐level pillar surface, these liquids flow independently onto the top of the pillars, and liquid bridges gradually form on each pillar with a uniform area (**Figure** **2**a). This is significantly different from a continuously flowing liquid film on a smooth surface (Figures S5a and S6a, Supporting Information). This unique liquid uniform distributing process induced by pillar arrays is regarded as self‐splitting. While on the bioinspired hierarchical pillar surface, self‐splitting occurs in turn on first‐level pillars and second‐level pillars in the same manner to form a more uniform liquid distribution. Finally, two‐level liquid self‐splitting automatically occurs with the continuous decline of liquid and gap distance *d*, i.e., Δ*d* > 0 (Figure 2b; Movie S2, Supporting Information). As for micro–nano hierarchical pillars on tree frog's toe pads, many more uniform thin liquid films in nanoscale could be generated than for single‐level micropillars or smooth surfaces.

To achieve uniform thin films on the pillars’ tops, it is vital to make self‐splitting occur as much as possible during the FLC steps. During the separation of contact surfaces, liquid is continuously extracted from channels onto pillar tops and uniformly split into numerous liquid bridges by the pinning of pillar edges (Figure 2c; Movie S3, Supporting Information). This extraction effect mainly results from the capillary pressure of the narrow gap distance *d* being stronger than that of the wide channel width *w* (Figure 2d).^[^ ^27^ ^]^ Here, define *V*_P_ as the theoretical available liquid volume on each pillar when *d* = *w*, and *V*_C_ as the liquid volume in the channels around a pillar at *d* = 0 (Figure 2e). Generally, the liquid in the channels first flows onto the pillar tops as the gap distance *d* increases from 0 to *w*, and then redistributes when *d* exceeds over *w*. When *V*_P_ < *V*_C_, liquid in channels cannot be completely drawn out and is still connected together; thus, the liquid on the pillar tops flows back to the channels when *d* exceeds *w*, and self‐splitting fails (Figure 2g, *V*_P_: *V*_C_ = 0.7; Figure S7a and Section S1, Supporting Information). While *V*_P_ ≥ *V*_C_, all the liquid in the channels can be drawn out, and separate liquid bridges are generated on pillars under the liquid pinning effect in both the corner and side directions (Figure 2f,g, *V*_P_: *V*_C_ = 1.0–3.1; Figure S6b, Supporting Information).^[^ ^28^ ^]^ However, when *V*_P_ is much larger than *V*_C_, smaller liquid bridges form on the large pillars, which greatly decreases the area of capillarity. Therefore, the pillar arrays surface with *V*_P_ ≈ *V*_C_ is ideal for the formation of liquid self‐splitting effect to form a more uniform liquid distribution and higher area of capillarity. This *V*_P_ ≈ *V*_C_ feature is similar to the structural size of the tree frog's toe pad.

### 2.3 Nanocharacterization of the Liquid Rim Self‐Sucking Effect

**Figure 3:**
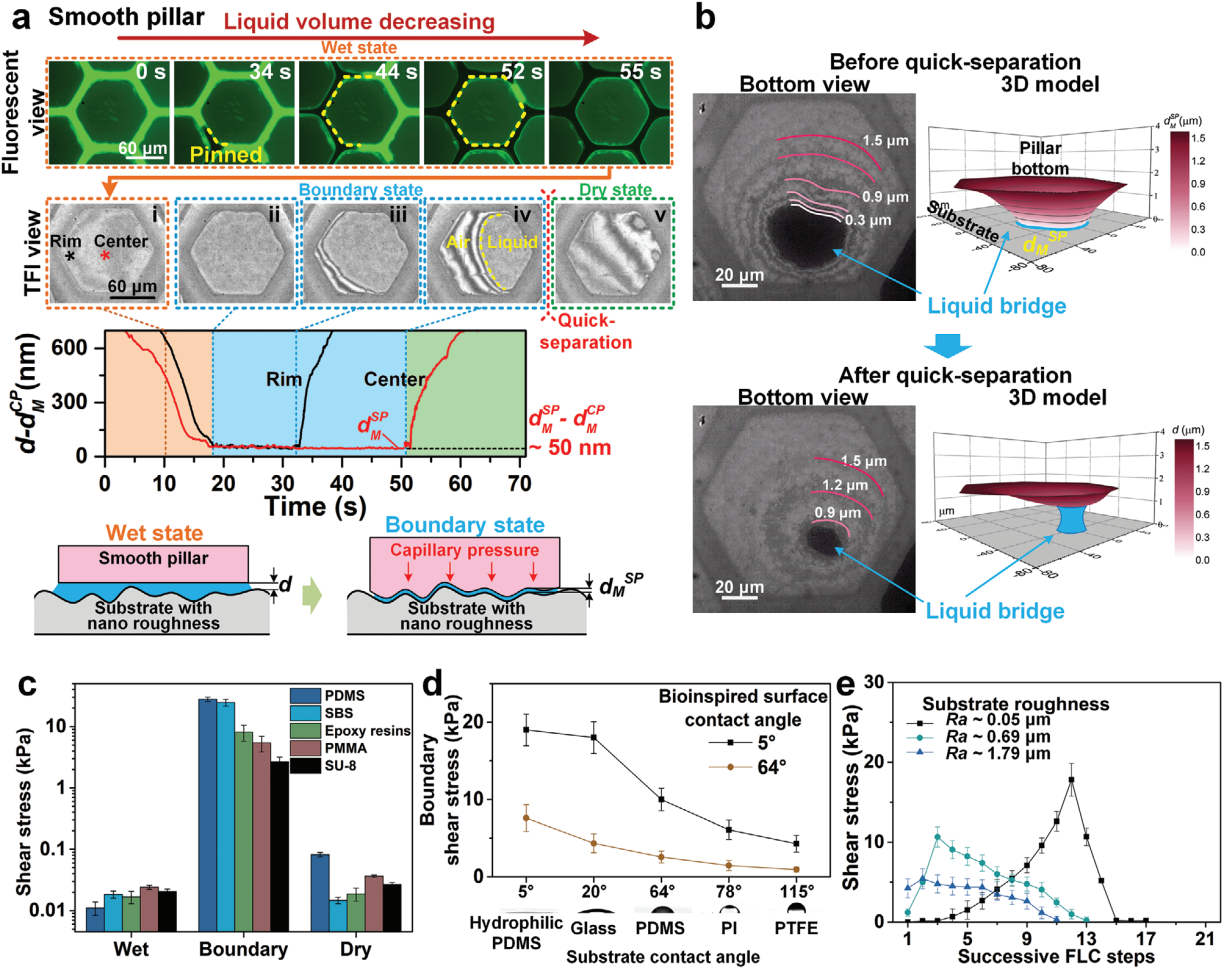
Strong boundary friction formed by nanometer‐thick liquid film. a) During evaporating, fluorescence observation shows that liquid is evaporated away from channels and self‐splitting forms on pillars. With thin film interference (TFI; Figure S8a, Supporting Information) view, solid contact in nanometer thick has been observed between pillar and smooth glass substrate (roughness, ≈50 nm) at boundary state. *d*_M_^SP^ and *d*_M_^CP^ are the minimum gap distance of smooth pillar and concave pillar, respectively. b) The deformation of pillar bottom can be clearly depicted by the TFI fringes. Since liquid volume remains almost unchanged during quick‐separation (Figure S8b, Supporting Information), its thickness before quick‐separation *d*_M_^SP^ is calculated by dividing the liquid volume, obtained from liquid sectional area and thickness after quick‐separation, by sectional area before quick‐separation. It is calculated to be ≈ 200 nm by recording quick‐separation of ten pillars. c) After plasma hydrophilic modification, strong boundary friction could be generated on various materials fabricated bioinspired pillar surfaces. d) Hydrophilicity for tested surfaces and substrates are crucial for generating stronger boundary friction. e) Rougher substrates lead to lower boundary friction. Data are presented as mean ± SD and sample size *n* = 10.

Self‐splitting generates uniform liquid nanometer‐thick films in large area, which can result in a strong capillary force to deform soft pillars. Fluorescent observations were conducted to demonstrate the flow of liquid around pillar, and thin film interference (TFI, 600 nm monochromatic light) was used to in situ observe the change of gap distance *d* in nanoscale resolution (Figure S8a and Section S2, Supporting Information,).^[^ ^29^, ^30^ ^]^ To stably observe the liquid capillarity effect, the liquid volume was decreased by evaporation. During liquid evaporating from wet to near dry on a smooth pillar, the liquid initially decreases from the channels while the interference fringes on the pillar remain steady, indicating that the gap distance *d* is mostly unchanged (**Figure** **3**a, fluorescence view; Figure S7b, 0–31 s, and Movie S4, Supporting Information). After the liquid automatically self‐split and completely vanished from the channels, the gap distance *d* begins to decrease as the bright‐dark interference fringes move from pillar center to edge. The fringes eventually turn into a uniform light intensity with *d* reaching its minimum value *d*_M_^SP^ and the pillar enters the boundary state (Figure 3ai,ii; Figure S7b, 38–43 s, and Movie S5, Supporting Information). With further evaporating, the liquid film begins to shrink and the pillar gradually separates from its edge, until a sudden quick separation occurs between the pillar and substrate (Figure 3aiii–v; Figure S8b and Movie S6, Supporting Information). The diagram of the gap distance *d* clearly demonstrates this approaching‐separating process (Figure 3a, lower; Figure S9a, Supporting Information). The decrease of the gap distance *d* generates a growing capillary force until the pillar enters a solid contact to the substrate with *d*_M_^SP^ (Section S3, Supporting Information). The *d*_M_^SP^ is experimentally estimated to be ≈200 nm for a hydrophilic‐treated soft PDMS surface (Figure 3b; Movie S6, Supporting Information), and this nanometer‐thick water film can generate ≈700 kPa capillary pressure, much higher than a vacuum sucker with the highest 1 atm pressure. Such self‐formed strong capillary force could push the pillar against the smooth substrate firmly to form nano asperities interlocks (Figure S12a, Supporting Information), thus generating powerful boundary friction with a shear stress ≈20 kPa even without an external or preloaded normal force (Figure 1j,k; Movie S7 and Section S3, Supporting Information). The nanometer‐thick liquid film generated strong boundary friction is also validated by the deformation of three types of pillar under lateral force (Figure S10, Supporting Information).

During the FLC tests with liquid gradually decreasing, liquid bridges with strong capillarity start to appear on some pillars to generate boundary friction, and the surface turns from wet to hybrid friction. As the number of boundary pillars gradually increases, the hybrid friction progressively increases (Figure S11a,c, Supporting Information). Eventually, owing to the uniform liquid distribution induced by the self‐splitting effect, the majority of pillars enter the boundary state, and the maximum boundary friction is achieved. The steps number of hybrid friction and boundary friction on the bioinspired hierarchical pillar surface are much higher than those on bioinspired single‐level pillar surface or smooth surface (Figure S11b, Supporting Information). This strong friction maintaining ability from the hierarchical pillars structure has allowed tree frogs to adapt diverse wet living environments. Remarkably, such strong boundary friction also appears on different materials (Figure 3c); however, its strength is strongly affected by contact surface's material properties, including wettability, roughness, and elastic modulus. Lower hydrophilicity results in lower boundary friction (Figure 3d), which offers a boundary friction control strategy.^[^ ^31^ ^]^ Rougher substrates or harder pillar surfaces also decreases the boundary friction because both lead to thicker liquid film and weaker capillarity (Figure 3e; Figures S8b–d and S11a, Supporting Information).

**Figure 4:**
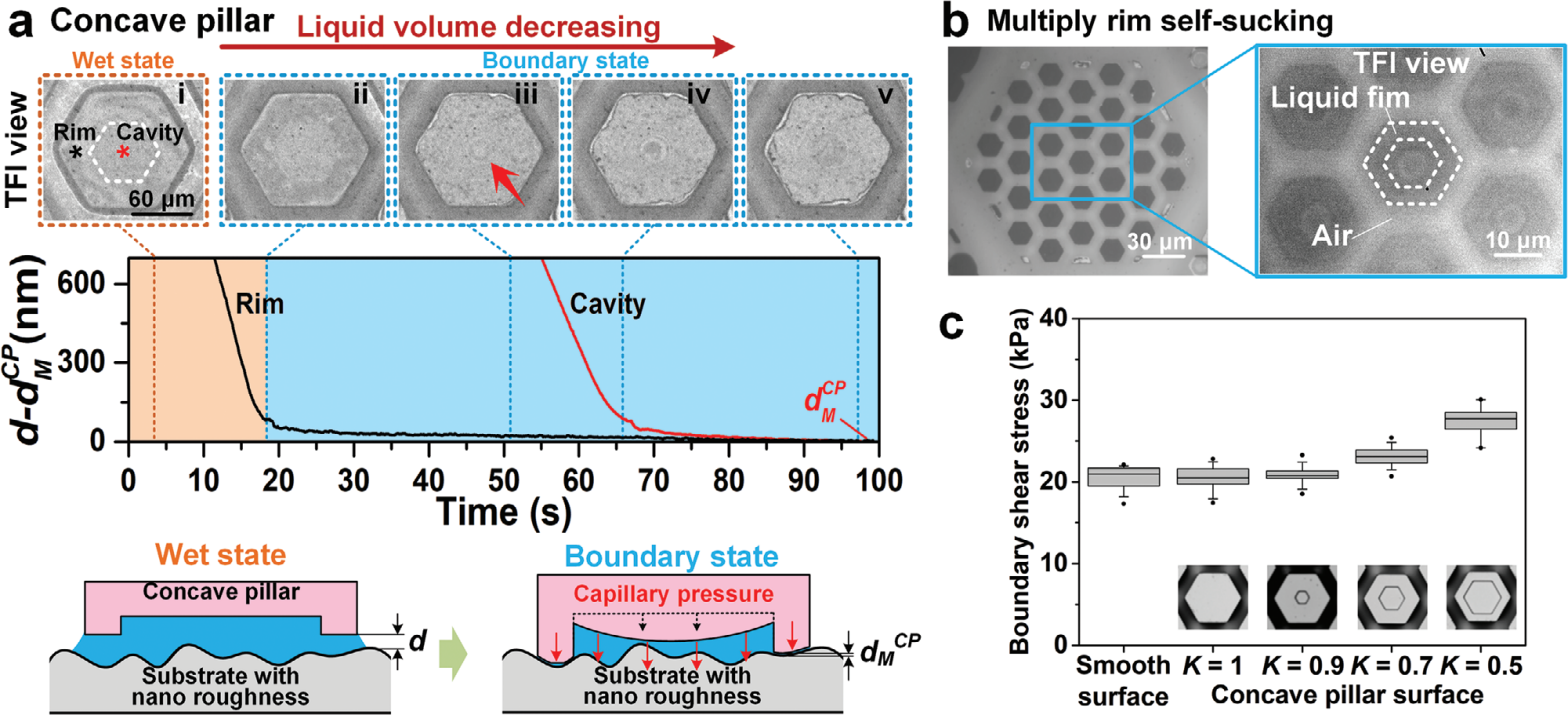
Boundary friction enhancing of rim self‐sucking effect. a) On concave pillar, liquid film area remains unchanged during evaporating as liquid in cavity continuously flows to rim area. The cavity bottom is gradually pulled to substrate and forms rim self‐sucking effect. Gap distance at rim of concave pillar *d*_M_^CP^ is ≈50 nm thinner than *d*_M_^SP^ on smooth pillar. b) On bioinspired hierarchical concave pillar surface, liquid self‐splitting effect could induce multiply rim self‐sucking simultaneously. c) Concave pillar surfaces demonstrate stronger boundary friction than smooth surface. With ratio of rim area to pillar area *K* gradually decreasing, stronger self‐sucking effect forms and generates stronger boundary friction. No significant difference shows among smooth surface and pillar surface with *K* = 1, 0.9 under one‐way ANOVA with *p* = 0.8. While significant difference shows for surface with *K* = 0.9–0.5 under one‐way ANOVA with *p* < 0.05 (sample size *n* = 10).

Under the inspiration of nanocavities on the tree frog's toe pads, concave pillars could generate an even stronger capillarity via a self‐sucking effect (**Figure** **4**a, upper). With continuous evaporation, the solid contact first forms around the rim area of the concave pillar instead of over the entire area, as with the smooth pillar (Figure 4ai,ii; Figure S12 and Movie S5, Supporting Information), and the liquid in the cavity is continuously pumped into the rim. By such powerful capillary pressure, the cavity bottom is gradually stretched toward the substrate (Figure 4aii–iv). This process further squeezes the gap distance at rim area to creates an even stronger capillary pressure in turn (Figure 4a, lower), which automatically forms the rim self‐sucking effect. Eventually, the minimum gap distance at rim *d*_M_^CP^ appears when the cavity bottom has been stretched to contact with the substrate (Figure 4av; Section S3, Supporting Information). The *d*_M_^CP^ is measured ≈50 nm thinner than *d*_M_^SP^ on smooth pillar (Figures 3a and 4a, lower; Figure S9, Supporting Information), which could generate stronger capillary pressure and maintain longer boundary state. Correspondingly, both the boundary friction and its endurance on concave pillar could be further enhanced by the self‐sucking effect (Figure 4c; Figure S13, Supporting Information). In particular, the hierarchical pillars induced two‐level liquid self‐splitting effect could benefit the simultaneous formation of rim self‐sucking on concave pillars as much as possible (Figure 4b).

### 2.4 Theoretical Mechanics for Friction Enhancement by Self‐Splitting and Self‐Sucking Effects

These automatically occurring self‐splitting and self‐sucking effects can synergistically enhance wet friction. Compared with a smooth surface, the friction enhancing coefficient of the hierarchical concave pillars *ζ*_F_(*K*, *N*) is affected by two structural features, including the ratio of the rim area to the pillar area on concave pillars, *K*, and the number of pillars related to the substrate roughness, *N*, which can be given by

(1) $$\zeta_{F}\;\left(K,\;N\right)=\zeta_{\mathrm{Sk}}\left(K\right)*\zeta_{\mathrm{Sp}}\left(N\right)$$

where *ζ*_Sk_(*K*) and *ζ*_Sp_(*N*) are the enhancing coefficients of the self‐sucking and self‐splitting effects, respectively. Here, the self‐sucking enhancing coefficient *ζ*_Sk_(*K*) can be derived as the ratio of the boundary friction on concave pillars to that on smooth pillars, which is

(2) $$\zeta_{\mathrm{Sk}}\left(K\right)=\;1+\frac{E^{\prime }*{\left(\sigma /\beta \right)}^{\frac{1}{2}}*\left[d_{M}^{\mathrm{SP}}-d_{M}^{\mathrm{CP}}\left(K\right)\right]}{\pi^{\frac{1}{2}}\gamma \left(\cos\theta_{S}+\cos\theta_{P}\right)+E^{\prime }*{\left(\sigma /\beta \right)}^{\frac{1}{2}}*d_{M}^{\mathrm{CP}}\left(K\right)}$$

where *E*′, *γ*, *θ*_S_, and *θ*_P_ are the effective elastic modulus, liquid surface tension, substrate, and pillar intrinsic contact angle, respectively; *η*, *σ*, and *β* denote the density of the asperities, the standard deviation of the height distribution and the radius of the asperity summits of the substrate surface, respectively. Since $d_{M}^{\mathrm{CP}}\left(K\right)$ declines from $d_{M}^{\mathrm{SP}}$ with *K* decreasing from 1 (Equation (S10) and Section S3, Supporting Information), *ζ*_Sk_ is always larger than 1 and increases with a decreasing *K*, as shown in Equation (2) (Figure S14, Supporting Information), which indicates a thinner rim of the pillar cavity generating a stronger self‐sucking effect. Specifically, taking a typical smooth glass slide with nanoroughness as a substrate, the theoretical results demonstrate that decreasing *K* from 1 to 0.5 can lower $d_{M}^{\mathrm{CP}}$ by ≈30%, thus increasing *ζ*_Sk_ by ≈27% (Table S2, Supporting Information). This agrees well with the measured friction on different concave pillar surfaces, with the friction rising by ≈33% as *K* decreased from 1 to 0.5 (Figure 4c). With respect to pillar pattern as on the toe pad of the tree frog, *K* approximates to 0.4 and could even increase the boundary friction by ≈40%. Besides, the self‐sucking effect can also decrease liquid evaporating speed to length the boundary friction duration (Section S4, Supporting Information). For example, on a concave pillar with *K* ≈0.65, the boundary friction duration can be enhanced up to ≈4 times longer than that for a smooth pillar (Figure S13 and Movie S8, Supporting Information). The duration could be further enhanced by as much as ≈30 times for the toe pad of the tree frog, with *K* ≈0.4 (Table S2, Supporting Information).

**Figure 5:**
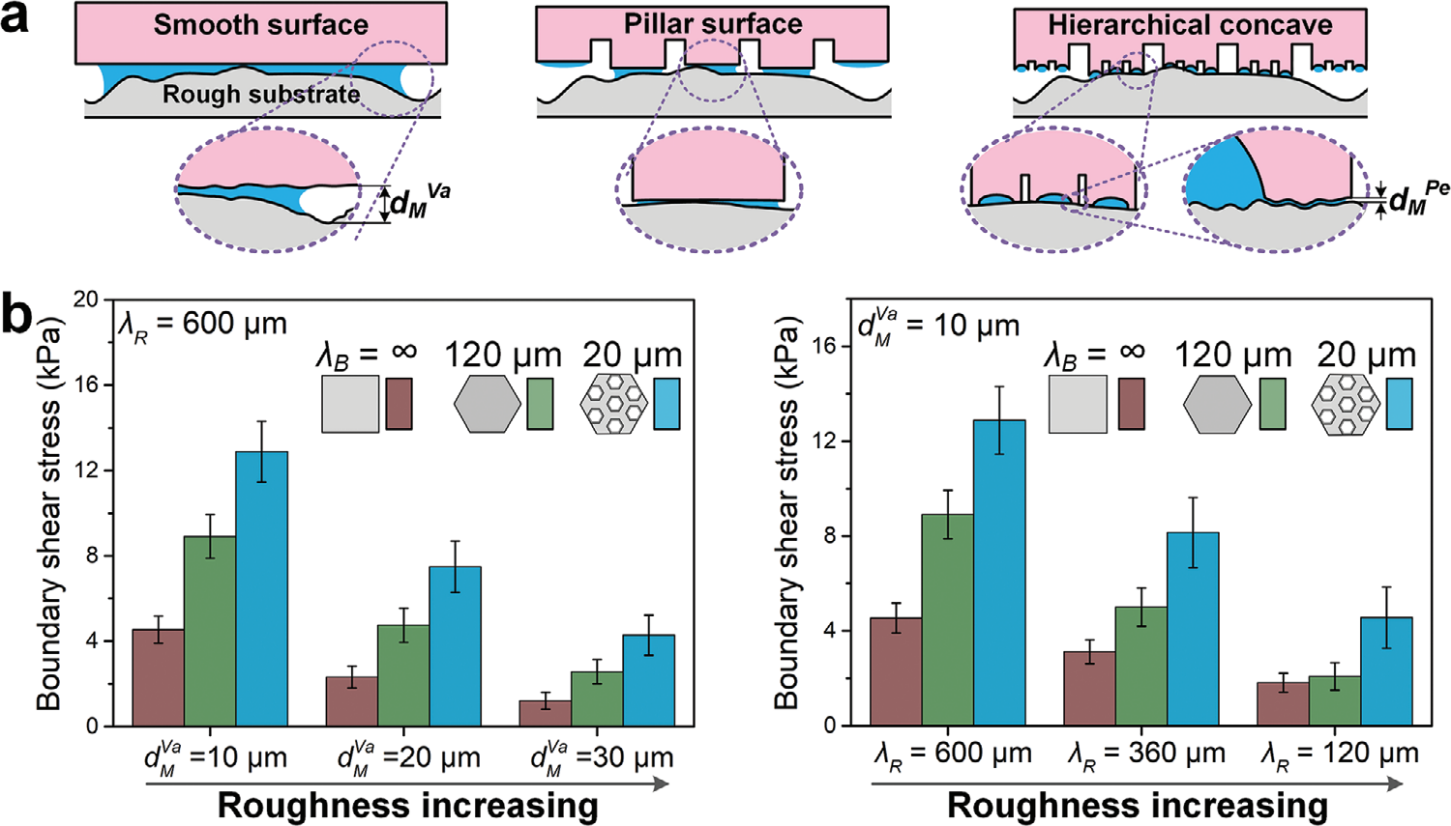
Boundary friction enhancing of liquid self‐splitting effect. a) When contact with rough substrate, bioinspired hierarchical pillar surface under the help of self‐splitting effect forms more and thinner liquid films than on smooth surface, which leads to much lower boundary friction. *d*_M_^Pe^ and *d*_M_^Va^ represent the gap distance at roughness bump peak and bump valley. b) The boundary friction of smooth, bioinspired single‐level pillar and bioinspired hierarchical concave pillar surfaces are tested on various rough substrates with different *d*_M_^Va^ and *λ*_R_. *λ*_R_ is the rough wavelength of substrate (Figure S16a,b, Supporting Information). *λ*_B_ is the wavelength of bioinspired surface, i.e., the diameter of a pillar. Under the enhancing effect of self‐splitting, bioinspired pillar surfaces generate much stronger boundary friction than smooth surface. Data are presented as mean ± SD; sample size *n* = 10.

When a smooth surface contacts a rough substrate, liquid is prone to gather into large droplets and form thicker liquid bridges around the deep roughness valleys (**Figure** **5**a, left). On bioinspired pillar surfaces, the self‐splitting effect distributes liquid more uniformly and forms many more nanometer‐thick liquid film patches around roughness bumps, generating stronger capillarity and enhanced friction (Figure 5a, middle and right). Simplifying the rough substrate to a spherical bump arrays surface, the enhancing coefficient of the self‐splitting effect *ζ*_Sp_(*N*) can be described as the ratio of the boundary friction on a bioinspired pillar surface to that on a smooth surface, which is

(3) $$\begin{array}{ccc} & & \zeta_{\mathrm{Sp}}\;\left(N\right)\\ & & =\sum_{n=1}^{N}\frac{2\left(2n-1\right)\hat{D}}{\left[\hat{D}+1+\frac{{\hat{\Lambda }}^{2}}{4\left(\hat{D}-1\right)}-\sqrt{{\left(\hat{D}-1+\frac{{\hat{\Lambda }}^{2}}{4\left(\hat{D}-1\right)}\right)}^{2}-{\left(\frac{n}{N}*\hat{\Lambda }\right)}^{2}}\right]N^{2}} \end{array}$$

where $\hat{D}$ and $\hat{\Lambda }$ are the normalization of the minimum gap distance at the bump valley $d_{M}^{\mathrm{Va}}$ and the substrate roughness wavelength *λ*_R_ to the minimum gap distance at the bump peak $d_{M}^{\mathrm{Pe}}$, respectively, that is, $\hat{D}=d_{M}^{\mathrm{Va}}\;/d_{M}^{\mathrm{Pe}}$ and $\hat{\Lambda }=\lambda_{R}\;/d_{M}^{\mathrm{Pe}}$; *N* refers to the number of pillars covering half of the cross section of a bump, *N*  = *λ*_R_ /2*λ*_B_, where *λ*_B_ is the pillar diameter of the bioinspired surface (Figures S16a,b and S17 and Section S5, Supporting Information). When *N* is increased by reducing the pillar diameter *λ*_B_, the proportion of pillars with a lower gap distance is increased by the self‐splitting effect to give a stronger capillary force, thus increasing *ζ*_Sp_(*N*) (Figure S18, Supporting Information). In addition, *ζ*_Sp_ increases larger as the substrate becomes rougher, with higher $d_{M}^{\mathrm{Va}}$ and smaller *λ*_R_ (Figure S18b,c, Supporting Information).^[^ ^32^ ^]^ Therefore, the self‐splitting effect allows bioinspired pillar surfaces to generate strong boundary friction more easily and makes the surfaces more suitable for contact with a rough substrate. For example, for a bioinspired pillar surface with *λ*_B_ = 200 nm to contact a rough substrate with $d_{M}^{\mathrm{Pe}}$ = 100 nm, $d_{M}^{\mathrm{Va}}$ = 1 µm, and *λ*_R_ = 20 µm, *ζ*_Sp_ is as high as 7.2 for *N* = 100 (Section S5, Supporting Information). Friction tests on rough substrates have validated the self‐splitting enhancing effect, and the boundary friction of these pillar surfaces is much higher than that for smooth surfaces (Figure 5b). With respect to the hierarchical pillar surface (*N* = 15), the boundary friction can be ≈3.5 times higher than that of a smooth surface. The measured self‐splitting enhancing coefficient, *ζ*_Sp_, agrees well with the theoretical value (Figure S16c,d, Supporting Information). Due to the cooperative effects of self‐splitting and self‐sucking, the bioinspired concave pillar surface generates the highest boundary friction.

### 2.5 Applications of Bioinspired Strong Wet Friction Surfaces

**Figure 6:**
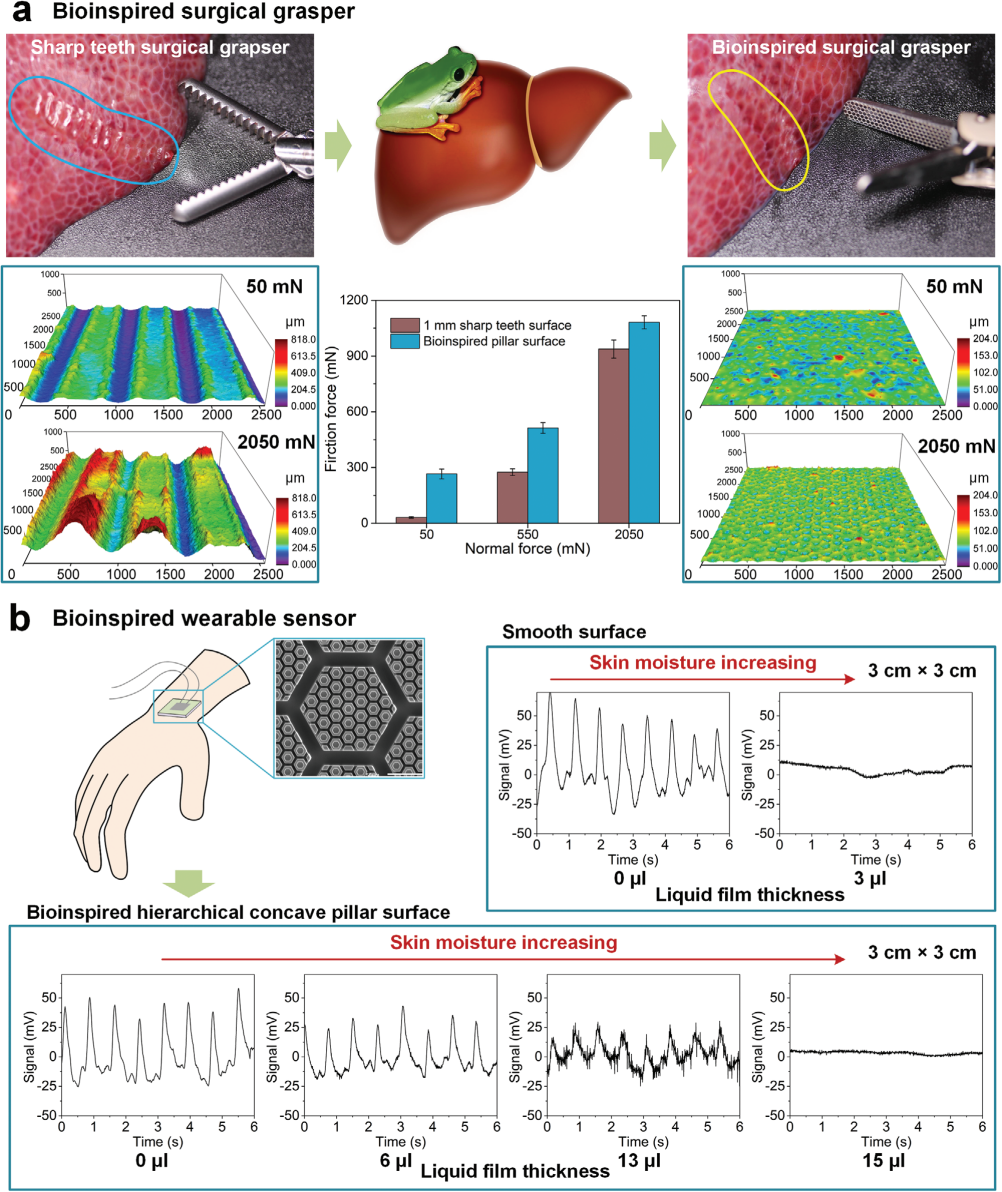
Applications on biomedical devices and wearable flexible electronics. a) The tissue deformation and friction performance (sample size *n* = 5) have been characterized on 1 mm sharp teeth surface and bioinspired hierarchical concave pillar surface (Figure S19a,b, Supporting Information). Based on the safety grasping threshold of pig liver, normal load of 50, 550, and 2050 mN were applied during friction measurement. Bioinspired surface shows much lower tissue deformation and stronger friction than modern sharp teeth surface. b) On smooth surface (area 3 cm × 3 cm), moist skin leads to weak attachment and pulse signal cannot be distinguished. While on bioinspired hierarchical concave pillar surface, the pulse signal is still distinguishable even with ≈13 µL liquid film on skin (Figure S19c,d, Supporting Information).

Bioinspired pillar surface has diverse potential applications in wet biointerface contact such as wearable flexible electronics, implant devices, precision medicine and biomedical engineering due to liquid always exists on surface of biological tissue (**Figure** **6**). Particularly, modern sharp teeth surgical graspers need a strong external force to deform the tissue to form a secure grasp, which can easily lead to soft tissue damage. By applying the bioinspired pillar surface on the surgical grasper, the friction can be as high as ≈5 times of its normal force and ≈9 times of the friction on the sharp teeth surface, while the deformation of soft tissue can be decreased by 90% less than that of sharp teeth surface, which can effectively reduce tissue damage (Figure 6a). Even for wearable flexible electronics, signal distortion is unavoidable when the skin is sweaty (Figure 6b, upper right). By applying bioinspired hierarchical concave pillar surface to wearable sensor, the sweat tolerability can be improved by ≈5 times compared with a smooth surface (Figure 6b, lower).

## 3 Conclusion

In summary, a strong wet attachment surface has been proposed under the inspiration of tree frog toe pad's superior wet attachment even without special external or preloaded normal force. The unique hierarchical surface structures of the toe pads, i.e., micro–nano hierarchical pillars and nanocavities existing on the top of the second‐level pillars, are extracted as the typical structural features for the bioinspired surfaces. With liquid movement observation, a two‐level self‐splitting effect is found to uniformly distribute liquid on the pillars, and a self‐sucking effect appears automatically with the cavity rims to form a thinner nanometer‐thick liquid film. Both the self‐splitting and self‐sucking effects improve the possibility of strong capillary liquid bridge formation, which lead to the formation of stronger boundary friction. Combining these two effects, the boundary friction on bioinspired concave pillars could be improved over 3.5 times as compared with a smooth surface. This work further expands the soft material friction theory and can be applied to climbing robots, medical devices, and wearable electronic devices.

## 4 Experimental Section

##### Sample Preparation and Structural Characteristics of Tree Frog Toe Pad

Tree frogs, *R. dennysi* (sample size *n* = 3), obtained from a local supplier, were raised in a terrarium and fed with water and crickets. After washing and cleaning with 0.1 mol L^−1^ phosphate‐buffered solution, the toe specimens of frogs were immersed into 2.5% glutaraldehyde at pH 7.4 for 4 h, then washed with 0.1 mol L^−1^ phosphate‐buffered solution three times for each time lasting 15 min, and postfixed in 1% osmium tetroxide buffered solution for 1 h. After dehydrated in graded ethanol and immersed in tertiary butyl alcohol for 15 min, the specimens were finally dried in a vacuum freeze drier for 24 h.^[^ ^33^, ^34^ ^]^ A scanning electron microscope (JSM‐7500, Japan Electronic) and atomic force microscope (Dimension Icon, Bruker) were applied to observe surface morphology. All the animal protocols were approved by the Biological and Medical Ethics Committee of Beihang University with authorization number of BM20170087.

##### In Situ Observation of Mucus Secreting on Tree Frog's Toe Pad

Tree frog was washed clean with deionized water and left on dust‐free paper for 20 min. The observation was operated under a microscope (BX51, Olympus). 2 µL Chinese ink (water mixed with carbon nanoparticles) was filled onto the toe pad to distinguish channels and glands, and the excreting process of mucus could be displayed by the flow of Chinese ink.

##### Liquid Film Characteristics and Friction Tests for a Tree Frog Toe Pad during FLC Steps

A living tree frog was thoroughly washed and then anesthetized with Isoflurane gas (Hebei Jiupai Pharmaceutical Co.). Its toe pad was fixed to a glass tube with gentle vacuum pressure and moved by a motorized 3D‐translation stage (Figure S3b, Supporting Information). During successive FLC steps, the toe pad was lifted glass slide substrate and then put down on a new area of the substrate (one step). After each step, the friction of the toe pad was recorded with 2D force measuring equipment (minimum division value of 0.2 mN and max load of 10 000 mN), with a normal force of 5 mN and a sliding speed of 200 µm s^−1^, as shown in Figure S3c (Supporting Information). After the toe pad reached high steady hybrid friction during the FLC steps, the toe pad secretion was further decreased by wiping 10, then 20, times (*N*_1_ and *N*_2_) with dust‐free paper to produce boundary and dry friction conditions, respectively. The FLC tests were repeatedly performed ten times for toe pad of three alive tree frog at ambient temperature. The secretion film state between the contact areas was captured via a microscope with an attached high‐speed camera (X100, Photron), illuminated from below, to give a clear view of the liquid film (Figure S3d, Supporting Information).

##### Fabrication of Bioinspired Surfaces and Rough Substrates

A smooth surface (1 cm × 1 cm) was replicated from a glass slide by PDMS(Sylgard 184, Dow Corning). A bioinspired single‐level pillar surface (1 cm × 1 cm) and rough substrates were obtained by replicating SU‐8 (T2035, Micro Chem) models with PDMS (Figure S1a, Supporting Information. To fabricate the bioinspired hierarchical pillar surfaces, a silicon wafer was etched with small hexagonal pits using Si deep reactive ion etching (DRIE), and larger hexagonal pits were fabricated over the smaller hexagonal pits with SU‐8. The surface was finally prepared by PDMS replication (Figure S1b, Supporting Information). Various other materials were used to fabricate bioinspired hierarchical pillar surfaces by replicating from a PDMS mold, including the use of styrene–butadiene–styrene block copolymers (SBS), acrylonitrile butadiene styrene (ABS), polymethyl methacrylate (PMMA), epoxy resins, and photoresist SU‐8. To fabricate bioinspired hierarchical concave pillar surfaces, a silicon wafer was thermally oxidized to form a nanolayer of SiO_2_ on its surface. This SiO_2_ layer was patterned by photolithography to provide a more durable mask during Si DRIE and then covered by a layer of photolithographed SU‐8. Three‐level structures were fabricated successively, including first‐level microgrooves, second‐level microgrooves and third‐level microcavities. Each step was accompanied by the removal of SU‐8 or SiO_2_ reactive ion etching to form necessary masks. The bioinspired surfaces were produced via PDMS double replication (Figure S1c, Supporting Information). Their structural properties are shown and listed in Figure S2 and Table S1 (Supporting Information). Before the FLC tests, PDMS samples were repeatedly treated with oxygen plasma (P8C, Schwarze) until their stickiness was negligible. The wettability of the samples was adjusted by the duration of oxygen plasma treatment.

Five types of rough substrates were designed as round bump arrays with height of 10, 20, and 30 µm and diameter of 600, 360, and 120 µm, respectively. These substrates were fabricated by 3D printing method with resolution of 2 µm (BMF Precision Co.) (Table S3 and Figure S16a,b, Supporting Information). The height and diameter of the bumps can be regarded roughness parameters, i.e., bump valley depth *d*_M_^Va^ and rough wavelength *λ*_R_, respectively.

##### Liquid Film Characteristics and Friction Tests for Bioinspired Surfaces during FLC Steps

For bioinspired surfaces’ FLC tests, liquid film observations were made with the same equipment that was used for the tree frog toe pad analysis. Ten times of tests were repeatedly performed at ambient temperature, and before each test, the surfaces were modified to hydrophilic using oxygen plasma. A volume of 1.5 µL deionized water was injected onto the test surface. Then, the test surface was lifted from the substrate on one side with a constant speed of 1 mm s^−1^ to mimic the peeling gait of tree frog (Figure S3c and Movie S1, Supporting Information). The FLC steps were under a normal force of 5 mN and a sliding speed of 200 µm s^−1^. The shear stress was obtained by dividing the friction force by the bioinspired surface area of 1 cm × 1 cm.

The interfacial liquid movement during liquid self‐splitting was characterized by fluorescent observation using Fluorescein sodium mixture (Fluorescein sodium salt, Solarbio; Movie S3, Supporting Information). To study the affection of pillar size (*V*_P_ and *V*_C_) on liquid self‐splitting effect, a cover glass was placed over the bioinspired surface, and two PDMS spacers were placed between the interface to form a gap distance *d* higher than channels width *w* (Figure S7a, Supporting Information). The PDMS spacers were fabricated by a spin‐coating method and their thicknesses were controlled by adjusting the spin‐coating rotating speed. The liquid self‐splitting effect could be subsequently characterized by pressing and releasing the cover glass.

The pillars’ lateral deformation during FLC tests was characterized by a microscope (Figure S10a, Supporting Information). Dark areas denote the liquid between the pillar and the substrate. Pillars exhibited lateral deformation indicate that they were generating friction (Figure S10b–d and Movie S7, Supporting Information). Based on liquid distribution and pillar lateral deformation, these pillars were classified into dry, wet and boundary states during FLC tests, and the number of pillars in each state was counted as shown in Figure S11c (Supporting Information).

TFI were used to in situ characterize the gap distance and the interfacial liquid capillarity between pillars and substrate in boundary friction conditions. The bioinspired surface was adhered to a glass slide by liquid capillarity and placed facing up under a microscope with high‐speed camera (Figure S8a, Supporting Information). The light source used was monochrome with a wavelength of *λ* = 600 nm. To enhance the clearance of the TFI, a layer of chromium film with thickness of ≈10 nm was coated onto glass slide via magnetron sputtering. The transformation from TFI to gap distance is described in Section S2 (Supporting Information).

##### The Fabrication and Characterization of Bioinspired Surgical Graspers and Bioinspired Wearable Sensors

The prototype of stainless‐steel bioinspired surgical grasper was fabricated by chemical etching using designed hexagonal photoresist masking. Friction comparison tests were performed on PDMS fabricated bioinspired hierarchical concave pillar surface and 1 mm sharp teeth surface with area of 3 mm × 5 mm. Fresh pig liver was used as biological sample to perform a grasping test. The experiment setup is shown in Figure S19a,b (Supporting Information). Based on the safety grasping threshold of pig liver for ≈200 kPa,^[^ ^35^ ^]^ weights of 50, 550, and 2050 mN were separately added to these surfaces as normal load during friction measurement. All friction measurements were performed for five times at ambient temperature and data were presented as mean ± SD. The deformation of the pig liver was achieved by freezing the liver under different normal loads, then scanning with a laser microscope (Model OLS4100, Olympus Co.).

A bioinspired wearable sensor was made from medical silica gel (20‐40A, Wanzl Commercial Equipment Co.), which is the same material used in commercial skin adhesive. A piezoelectric sensor (from MEAS) made of polyvinylidene fluoride (PVDF) was embedded into the surfaces to act as a pulse sensor. During the test, the bioinspired wearable sensor and smooth surface sensor were attached to the skin of a person's wrist and different volumes of water (6, 13, and 15 µL) were added to the dry skin to mimic sweating conditions. The signals produced by the sensors were successively processed by a charge‐amplifier (26920S4, Bruel & Kjaer) and programmable filter (3642, NF ELECTRONIC INSTRUMENTS) and displayed on an oscilloscope (TDS 2012C, Tektronix) (Figure S19c,d, Supporting Information). All the human included experiments were performed according to IACUC guidelines and approved by the Biological and Medical Ethics Committee of Beihang University with authorization number of BM20170090. All the human subjects volunteered with informed consent.

##### Statistical Analysis

All tested data were carried out with Wolfram Mathematica (V10.1, Wolfram Research) and plot with OriginPro (V9.0, OriginLab Corp.). For bar diagrams, the data were presented as mean ± SD (standard deviation) and the sample size *n* was listed in corresponding figure legend. For the box plots in Figure 4c and Figure S11b (Supporting Information), the line in box represents median value, and the box represents 25% and 75% of data. The whiskers represent the 10% and 90% of data, and the dots represent max and min data. They were statistically analyzed by one‐way ANOVA with significant level *α* = 0.05.

## Conflict of Interest

The authors declare no conflict of interest.

## Supporting information

Supporting Information

Supplemental Movie 1

Supplemental Movie 2

Supplemental Movie 3

Supplemental Movie 4

Supplemental Movie 5

Supplemental Movie 6

Supplemental Movie 7

Supplemental Movie 8
