# Supplementary material for: Micro–Nano Hierarchical Structure Enhanced Strong Wet Friction Surface Inspired by Tree Frogs
Source: Adv Sci (Weinh). 2020 Aug 9;7(20):2001125. doi: 10.1002/advs.202001125 (PMC7578903; doi:10.1002/advs.202001125)
Supplement: Supplementary file 1 — Supporting Information [file ADVS-7-2001125-s001.pdf]

© 2020 Wiley-VCH GmbH

ADVANCED SCIENCE
Open Access

Supporting Information

for *Adv. Sci.,* DOI: 10.1002/advs.202001125

Micro–Nano Hierarchical Structure Enhanced Strong Wet Friction Surface Inspired by Tree Frogs

Liwen Zhang, Huawei Chen, Yurun Guo, Yan Wang, Yonggang Jiang, Deyuan Zhang, Liran Ma, Jianbin Luo, and Lei Jiang*

Copyright WILEY-VCH Verlag GmbH & Co. KGaA, 69469 Weinheim, Germany, 2020.

Supporting Information

Micro-nano hierarchical structure enhanced strong wet friction surface inspired from tree frog

Liwen Zhang, Huawei Chen, Yurun Guo, Yan Wang, Yonggang Jiang, Deyuan Zhang, Liran Ma, Jianbin Luo, Lei Jiang*

Dr. L. Zhang, Dr. Y. Guo, Dr. Y. Wang, Prof. Y. Jiang, Prof. D. Zhang

School of Mechanical Engineering and Automation

Beihang University

Beijing, 100191, China

Prof. H. Chen

School of Mechanical Engineering and Automation

Beihang University

Beijing, 100191, China

Beijing Advanced Innovation Center for Biomedical Engineering

Beihang University

Beijing, 100191, China

E-mail: chenhw75@buaa.edu.cn

Prof. L. Ma, Prof. J. Luo

State Key Laboratory of Tribology

Tsinghua University

Beijing, 100091, China

Prof. L. Jiang

Laboratory of Bioinspired Smart Interface Science

Technical Institute of Physics and Chemistry

Chinese Academy of Sciences

Beijing, 100190, China

List of symbols

α_V liquid vaporization coefficient

a side length of pillar

A_P, A_C area of pillar and capillary bridge

C_C circumference of capillary bridge

d, d_T gap distance and the thinnest gap distance between pillar and substrate

d_M^{SP}, d_M^{CP} minimum gap distance on smooth pillar and at rim of concave pillar

d_M^{Va}, d_M^{Pe} minimum gap distance at rough bump valley and bump peak

E, E' elastic modulus of tested surfaces and effective elastic modulus

F^{SP}, F^{CP} boundary friction of smooth pillar and concave pillar

F^{SS}, F^{BS} boundary friction of smooth surface and bioinspired surface on rough substrate

h pillar height

$I, \bar{I}$ light intensity and relative light intensity

I_{max}, I_{min} max and min light intensity

$\bar{I}_0^{Rim}$ relative light intensity of first level interference at the rim of concave pillar

k rate of cavity pressure to cavity volume

K ratio of rim area to pillar area on concave pillar

N number of pillars per rough bump along radius direction

P_C, P_E capillary pressure and elastic contact stress

P_{Con} pressure in cavity of concave pillar during evaporation

r radius of the spherical bump

s refractive index

t^{SP}, t^{CP} liquid evaporation time on smooth pillar and concave pillar

V_C volume of liquid in channels around a pillar

V_P maximum volume of liquid can be reserved on top of a pillar

V cavity volume on concave pillar during evaporation

w channel width between pillars

W_C, W_E capillary force and elastic contact force between a pillar and substrate

γ liquid surface tension

$\theta_P{}'$ apparent contact angle pillar

θ_S, θ_P intrinsic contact angle of substrate and pillar

η, σ, β density of asperities, standard deviation of the height distribution, and radius of asperity summits

ν Poisson's ratio of tested surfaces

μ frictional coefficient

λ_B diameter of pillar on bioinspired surface

λ_R wavelength of substrate roughness which can be represented by the sectional diameter of rough spherical bump

$\widehat{D}, \hat{\Lambda}$ normalization of d_M^{Va} and λ_R to d_M^{Pe}

$\delta(n)$ proportion of the nth liquid bridge

ζ_{Sk} boundary friction enhancing coefficient of self-sucking effect on concave pillar

ζ_{Sp} boundary friction enhancing coefficient of self-splitting effect on bioinspired pillar surface

ζ_F boundary friction enhancing coefficient on bioinspired concave pillar surface combining the self-sucking enhancing effect and self-splitting enhancing effect

Supplementary Text

1. Theoretical analysis of liquid self-splitting effect on bioinspired pillar surface

Since each channel belongs to two pillars, the volume of liquid in channels around each pillar which will be drawn onto the pillar top can be represented as

$$V_C = 3awh \tag{S1}$$

where a, w and h are the side length of pillar, the channel width, and the pillar height, respectively. The maximum volume of liquid can be reserved on top of each pillar is

$$V_P = \frac{3\sqrt{3}}{2} a^2 w \tag{S2}$$

The liquid self-splitting effect is influenced by

$$V_P : V_C = \frac{\sqrt{3}a}{2h} \tag{S3}$$

When $V_P < V_C$, liquid in channels cannot be all drawn out and gathered into a large liquid bridge, which leads to self-splitting failure. While $V_P : V_C$ is close to or higher than 1, self-splitting appears.

2. ***In-situ*** **observation of nano-liquid bridges on smooth pillar and concave pillar**

The bioinspired pillar surface is placed upside-down and adhered to a glass slide with 1.5 µL deionized water (Figure S8a). The microscope (BX51, Olympus) is set to reflect status to achieve thin-film interference between pillar and substrate with a wavelength λ = 600 nm light source. A high-speed camera (X100, Photron) is used to record the movement of interference fringes during water evaporation. To enhance the clearance of TFI, a layer of chromium film with thickness of ~10 nm is coated onto glass slide by magnetron sputtering. The light intensity of two points (~10 µm × 10 µm) on smooth pillar (at rim and center) and concave pillar (at rim and in cavity) have been achieved from one high-speed video simultaneously to allow the light intensity comparable.

Define relative light intensity as $\bar{I}$, which is

$$\bar{I} = \frac{2I - (I_{max} + I_{min})}{I_{max} - I_{min}} \tag{S4}$$

where I_{max} and I_{min} are the max and min light intensity at a point.[1] During liquid evaporating, pillar is gradually adhered to substrate with interference fringes continuously appearing from the center of pillar. When solid-solid contact forms with d decreasing to minimum gap distance d_M, a uniform light intensity appears on pillar, which forms the first level of interference. To make the gap distance on smooth pillar and concave pillar comparable, the change of TFI on both pillars are recorded at the same time in one shot. $\bar{I}$ of first level interference at the rim of concave pillar, $\bar{I}_0^{Rim}$, is higher than on smooth pillar, which means the minimum gap distance on concave pillar rim is thinner than on smooth pillar. Thus, minimum gap distance at concave pillar rim is regarded as the basic gap distance and represented as d_M^{CP}. The change of gap distance $d - d_M^{CP}$ on smooth pillar and concave pillar during evaporation can all be presented as

$$d - d_M^{CP} = \frac{\lambda}{4\pi s}\left[\left(n + \left|\sin\frac{n\pi}{2}\right|\right)\pi + \arccos(\bar{I})\cos n\pi - \arccos\left(\bar{I}_0^{Rim}\right)\right] \quad \text{(S5)}$$

where n = 0, 1, 2… and s is the refractive index of materials in gap; 1.33 for water and 1.00 for air.[1] The measured gap distance at rim and center of smooth pillar and concave pillar are shown in Figure S9.

3. Theoretical analysis of boundary friction increase induced by self-sucking enhancing effect

Based on capillary theory,[2] the capillary force between a pillar and substrate can be presented as

$$W_C(d, \theta_P{}') = P_C(d) * [A_P - A_P * K * M(d)]$$

$$= \frac{\gamma[A_P - A_P * K * M(d)](cos\,\theta_S + cos\,\theta_P{}')}{d} \quad \text{(S6)}$$

where P_C is capillary pressure, γ the surface tension of liquid, A_P the pillar area, ϑ_S and ϑ_P' are the intrinsic contact angle of substrate and apparent contact angle of pillar, respectively; d is the thickness of liquid bridge, i.e., the gap distance between interfaces; K is the ratio of rim area to pillar area on concave pillar (Figure S12). Specially, K = 1 is the situation on a smooth pillar. $M(d)$ is the real contact area ratio. If excessive liquid exists between the interfaces, W_C drops to almost zero when liquid spreads to the edge of pillar without any solid-solid contacts between interfaces, which results in wet friction. With liquid decreasing by FLC steps or evaporating, the capillary force gradually increases and drags surfaces into solid-solid contact and a resistant force is generated by its elastic contact deformation, which leads to boundary liquid film state and boundary friction (Figure S12). According to GW theory,[3] when the heights of rough substrate asperities follow an exponential distribution: $\phi^*(s) = e^{-s}$, the real contact area ratio $M(d)$ is

$$M(d) = \pi(\eta\beta\sigma)e^{-\frac{d}{\sigma}} \tag{S7}$$

where η, σ and β describe the surface properties of substrate, which are the density of asperities, the standard deviation of the height distribution, and the radius of asperity summits, respectively. Then, the elastic contact force W_E can be presented as

$$W_E(d) = P_E(d) * A_P * K * M(d) = \pi^{\frac{1}{2}}(\eta\beta\sigma)E'(\sigma/\beta)^{\frac{1}{2}}e^{-\frac{d}{\sigma}}KA_P \tag{S8}$$

Since the pillar is much softer than substrate, the effective elastic modulus $E' \approx E/(1-\nu^2)$, where ν is the Poisson's ratio and E the elastic modulus of pillar. During evaporating, the highest capillary adhesion $W_C(d_M, \theta_P)$ appears when d declines to the minimum gap distance d_M, and accompanied by θ_P' decreases to pillar's intrinsic contact angle θ_P (Figure S12). The force balance can be presented as

$$W_C(d_M, \theta_P) = W_E(d_M) \tag{S9}$$

On concave pillar, d_M is minimum gap distance at rim area since it forms solid-solid contact and liquid at rim edge determines the capillary pressure. Based on Equation S6, S8 and S9, the relationship between K and d_M is

$$K = \frac{\gamma(\cos\theta_S + \cos\theta_P)e^{\frac{d_M}{\sigma}}}{\gamma(\cos\theta_S + \cos\theta_P) * \pi(\eta\beta\sigma) + \pi^{\frac{1}{2}}(\eta\beta\sigma)E'(\sigma/\beta)^{\frac{1}{2}} * d_M} \tag{S10}$$

where d_M^{SP} can be achieved with K =1. $d_M^{CP}(K)$ decreases with K decreasing, which indicates larger cavity leads to thinner liquid film. In general, friction generated by solid-solid contact with normal force W_C can be described as

$$F = \mu W_C \tag{S11}$$

where μ is the frictional coefficient.[4,5] The self-sucking enhancing coefficient of boundary friction on concave pillar ζ_{Sk} can be presented as

$$\zeta_{Sk}(K) = \frac{F^{CP}}{F^{SP}} = \frac{W_C^{CP}}{W_C^{SP}} = 1 + \frac{E'(\sigma/\beta)^{\frac{1}{2}} * [d_M^{SP} - d_M^{CP}(K)]}{\pi^{\frac{1}{2}}\gamma(\cos\theta_S + \cos\theta_P) + E'(\sigma/\beta)^{\frac{1}{2}} * d_M^{CP}(K)} \tag{S12}$$

where F^{CP}, W_C^{CP} and d_M^{CP} are on concave pillar, and F^{SP}, W_C^{SP} and d_M^{SP} are on smooth pillar with K = 1.

For the case

$$\eta = 1000\ \mu\text{m}^{-2} \quad \sigma = 70\ \text{nm} \quad \beta = 30\ \text{nm} \quad E = 1.8\ \text{MPa} \quad \upsilon = 0.3$$

$$\gamma = 7.2 \times 10^{-2}\ \text{N/m} \quad \theta_S = \theta_P = 5°$$

the numerical solutions of d_M with different K are listed in Table S2. With K decreasing from 1 to 0.5, d_M decreases ~30%, and the boundary friction under self-sucking effect increases ~27%. It agrees well with the measured friction on different concave pillar surface that friction rises ~33% with K decreasing from 1 to 0.5 (**Figure 4c**).

Based on Equation S10 and S12, the increase of surface roughness (i.e. lower η, σ and β) can also lead to larger minimum gap distance d_M and weaker boundary friction, i.e. smaller ζ_{Sk}^{η}, ζ_{Sk}^{σ} and ζ_{Sk}^{β} (Figure S14). For a certain substrate, the elastic modulus E of bioinspired surface exhibits an optimum value that can generate the highest boundary friction (Figure S15). With surface roughness increasing, the optimum E decreases which indicates softer bioinspired surface is more suitable for rougher substrate. These results have also been proved by liquid film characterization (Figure S8b-d) and friction tests (Figure S11a and **Figure 3d,e**).

4. Theoretical analysis of boundary friction duration increase induced by self-sucking enhancing effect

Smaller minimum gap distance on concave pillar could also decrease the liquid evaporation, thus increasing the capillary bridge and boundary friction duration. On smooth pillar with liquid bridge evaporating from edge, the evaporating speed is considered to be proportional to its liquid film thickness d and liquid film circumference C_C. Define the liquid vaporization coefficient α_V, and the liquid evaporating speed can be presented as $C_C[1-M(d)]\alpha_V d$. The C_C has a relation to the liquid bridge area A_C, $C_C \approx 2\sqrt{\pi A_C}$. The evaporation time dt^{SP} for liquid bridge area decreasing dA_C is

$$dt^{SP} = -\frac{[1-M(d)]d}{C_C[1-M(d)]\alpha_V d} dA_C$$

For liquid bridge area A_C shrinking from pillar area A_P to 0, the evaporation time can be presented as

$$t^{SP} = \int_0^{A_P} -\frac{[1-M(d)]d}{C_C[1-M(d)]\alpha_V d} dA_C$$

$$= \int_0^{A_P} -\frac{dA_C}{2\sqrt{\pi A_C}\alpha_V} = \frac{\sqrt{A_P/\pi}}{\alpha_V} \tag{S13}$$

On concave pillar, the decrease of d_M and increase of real contact area ratio $M(d)$ lead to stronger sealing effect, which effectively reduces the liquid evaporation on concave pillar. Based on Equation S6 and S8, force equilibrium relationship during liquid evaporation is

$$W_C(d, \theta_P') = W_E(d) \tag{S14}$$

and the relationship between θ_P' and *d* can be obtained. The cavity deforming pressure P_{Con} results from capillary pressure P_C, and its relationship with cavity volume *V* can be simplified as

$$P_{Con} = P_0 + kV_0 - kV = P_C = \frac{\gamma(cos\,\theta_S + cos\,\theta_P')}{d} \tag{S15}$$

where P_0 and V_0 are the initial pressure and volume of cavity, and *k* the rate of cavity pressure to cavity volume, which can be achieved by

$$k = \frac{P_E(d_M) - P_0}{V_{Con}} \tag{S16}$$

Based on Equation S6, S8, S14 and S15, the relationship between *d* and cavity volume *V* during evaporating is

$$d = \sigma * \ln \frac{\pi(\eta\beta\sigma)\left[(P_0 + kV_0 - kV)K + \pi^{-\frac{1}{2}}E'(\sigma/\beta)^{\frac{1}{2}}K\right]}{P_0 + kV_0 - kV} \tag{S17}$$

The evaporation time on concave pillar is

$$t^{CP} = \int_{V_0}^{0} -\frac{1}{\alpha_V C_C[1 - M(d)]d}dV + \frac{\sqrt{A_P/\pi}}{\alpha_V}$$

$$= \int_{V_0}^{0} -\frac{1}{2\sqrt{\pi A_P}\alpha_V[1 - M(d)]d}dV + \frac{\sqrt{A_P/\pi}}{\alpha_V} \tag{S18}$$

Based on Equation S13 and S18, the boundary friction duration enhancing coefficient of self-sucking on concave pillar is

$$\zeta_{Sk}^{t} = \frac{t^{CP}}{t^{SP}} = \int_{V_0}^{0} -\frac{1}{2A_P[1-M(d)]d}\,dV + 1 \tag{S19}$$

For the case

$$A_P = 1.13 * 10^4\ \mu\mathrm{m}^2 \qquad P_0 = 0.01\ \mathrm{Mpa}$$

the numerical solutions based on Equation S16, S17 and S19 are shown in Table S2, and ζ_{Sk}^{t} increases with *K* decreasing. With *K* = 0.65,

$$\zeta_{Sk}^{t} \approx 4.3$$

which indicates the boundary states on concave pillar is ~4 times longer than on smooth pillar. It agrees well with the evaporation time on smooth pillar and concave pillar (Figure S13 and Movie S8). And this boundary friction duration can even be enhanced about 30 times higher as *K* = 0.4.

5. Theoretical analysis of boundary friction increase induced by self-splitting enhancing effect

Considering a smooth surface contact with a spherical rough bump on a rough substrate, liquid gathers around the bump and forms a thick liquid bridge with large minimum gap distance d_M^{Va} at bump valley (Figure S17a). Based on Equation S6 and S11, the friction generated by smooth surface on rough substrate F^{SS} can be described as

$$F^{SS} \approx \mu A_C P_C^{Va} = \mu A_C * \frac{\gamma(cos\,\theta_S + \cos\theta_P)}{d_M^{Va}} \tag{S20}$$

where A_C is the area of liquid bridge.[3-5]

When bioinspired surface contacts the bump, pillars at bump peak have much smaller minimum gap distance d_M^{Pe} (Figure S17b). The thickness of *nth* liquid bridge from bump center is

$$d_M(n) \approx d_M^{Pe} + r - \sqrt{r^2 - \left(\frac{n}{N} * \frac{\lambda_R}{2}\right)^2} \tag{S21}$$

where *N* is the number of pillars per rough bump along radius direction,[6] which can be presented as

$$N = \mathrm{int}[\frac{\lambda_R}{2\lambda_B}] \tag{S22}$$

where λ_R is the wavelength of substrate roughness, here can be regarded as the diameter of spherical bump section, and λ_B is the diameter of pillar. Specifically, *N* = 1 when λ_R is larger than $2\lambda_B$. d_M^{Va} can be represented as

$$r = \frac{d_M^{Va} - d_M^{Pe}}{2} + \frac{\lambda_R^2}{8(d_M^{Va} - d_M^{Pe})} \tag{S23}$$

where *r* is the radius of the spherical bump. The area proportion of the *nth* liquid bridge $\delta(n)$ is

$$\delta(n) = \frac{2n - 1}{N^2} \tag{S24}$$

The capillary pressure generated by the *nth* liquid bridge is

$$P_C(n) = \frac{\gamma(cos\,\theta_S + cos\,\theta_P)}{d_M(n)} \tag{S25}$$

The total friction generated by bioinspired concave pillar surface on rough substrate F^{BS} can be described as

$$F^{BS} \approx \mu * \sum_{n=1}^{N} [\delta(n) * A_C * P_C(n) * \zeta_{Sk}(K)]$$

$$= \zeta_{Sk}(K) * \mu A_C * \sum_{n=1}^{N} \frac{(2n-1) * \gamma(\cos\theta_S + \cos\theta_P)}{\left[d_M^{Pe} + r - \sqrt{r^2 - \left(\frac{n}{N} * \frac{\lambda_R}{2}\right)^2}\right] N^2} \quad \text{(S26)}$$

Define $\zeta_{Sp}(N)$ as the friction enhancing coefficient of self-splitting effect, which is

$$\zeta_{Sp}(N) = \sum_{n=1}^{N} \frac{(2n-1) d_M^{Va}}{N^2\left[d_M^{Pe} + r - \sqrt{r^2 - (n/N * \lambda_R/2)^2}\right]}$$

$$= \sum_{n=1}^{N} \frac{2(2n-1)\widehat{D}}{\left[\widehat{D} + 1 + \frac{\hat{\Lambda}^2}{4(\widehat{D}-1)} - \sqrt{\left(\widehat{D} - 1 + \frac{\hat{\Lambda}^2}{4(\widehat{D}-1)}\right)^2 - \left(\frac{n}{N} * \hat{\Lambda}\right)^2}\right] N^2} \quad \text{(S27)}$$

with the normalization by d_M^{Pe}: $\widehat{D} = d_M^{Va}/d_M^{Pe}$, $\hat{\Lambda} = \lambda_R/d_M^{Pe}$, $\widehat{D}_M(n) = d_M(n)/d_M^{Pe}$. With the number of pillars *N* increasing, the proportion of thinner capillary bridges increases (Figure S18a), thus increasing self-splitting enhancing coefficient $\zeta_{Sp}(N)$ (Figure S18b,c). Besides, $\zeta_{Sp}(N)$ increases with the increasing of $\widehat{D}$ and the decreasing of $\hat{\Lambda}$. With d_M^{Pe} = 100 nm, d_M^{Va} = 1 µm, λ_R = 20 µm and λ_B = 200 nm, the ζ_{Sp} can be as high as 7.2 for *N* = 100.

Finally, based on Equation S12, S26 and S27, we have the friction enhancement on bioinspired concave pillar surface combining the self-sucking enhancing effect and self-splitting enhancing effect,

$$\zeta_F(K,N) = \frac{F^{BS}}{F^{SS}} \approx \zeta_{Sk}(K) * \zeta_{Sp}(N) \quad \text{(S29)}$$

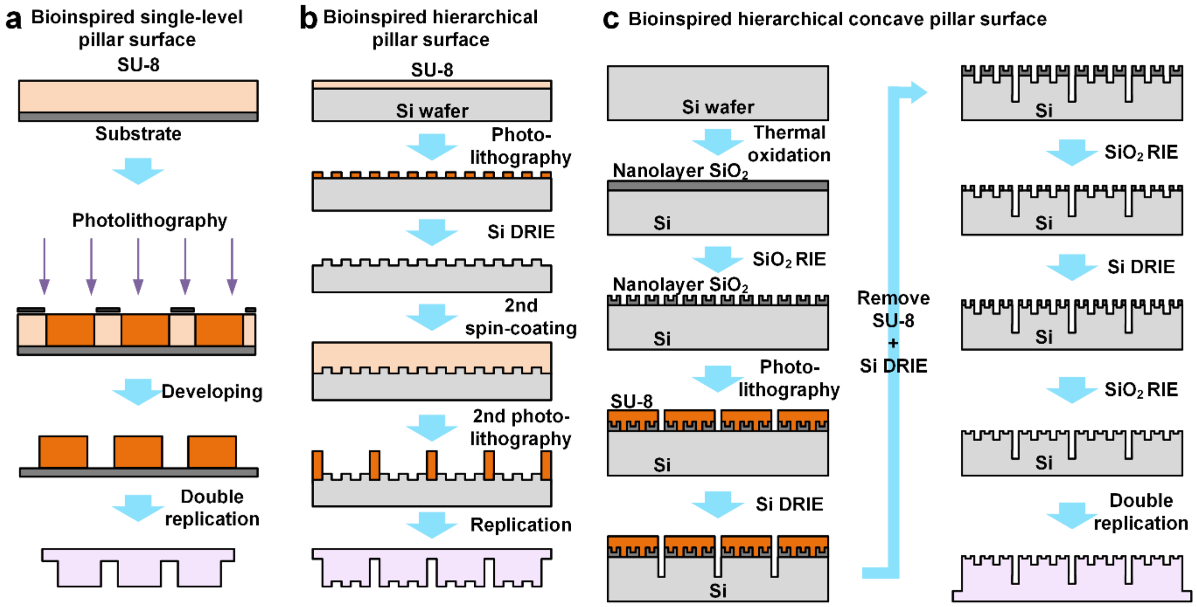

Figure S1. Bioinspired pillar surface fabrication procedures. (a) Bioinspired single-level pillar surfaces were replicated with PDMS by SU-8 models achieved from photolithography. (b) The SU-8 model of bioinspired hierarchical pillar surface is fabricated from two steps of photolithography, including 1st step to create smaller hexagonal pillar pits on Si water and 2nd step to build larger hexagonal pillar pits over these smaller cavities. (c) To fabricated bioinspired hierarchical concave pillar surface, a structured nanolayer SiO_2 is fabricated on Si wafer by photolithography and SiO_2 reactive ion etching (RIE). With a photoresist layer and SiO_2 nanolayer working as masks, the bioinspired hierarchical concave pillar surface is achieved after three times of Si deep reactive ion etching (DRIE). Then the PDMS surface is obtained by double replication.

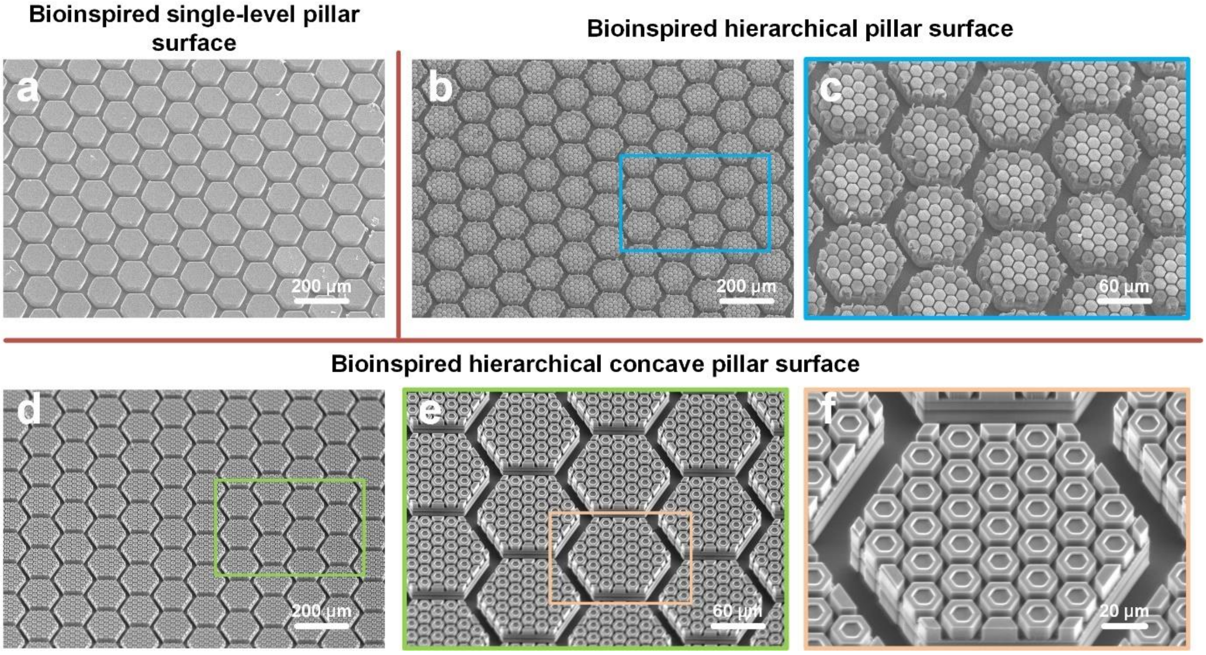

Figure S2. SEM images of bioinspired hierarchical pillar surface and bioinspired hierarchical concave pillar surface. (a) On bioinspired single-level pillar surface, the diameter of pillar is ~120 μm. The width and height of channels are 20 and 30 μm, respectively. (b,c) On bioinspired hierarchical pillar surface, the first-level micropillar has the same structure size as on bioinspired single-level pillar surface. The second-level pillar has a diameter of 20 μm. Width and height of second-level channels are 3 μm and 10 μm, respectively. (d-f) Bioinspired hierarchical concave pillar surface is constructed with hexagonal cavity on second-level pillar of bioinspired hierarchical pillar surface. The cavity is designed with diameter of 6, 12, 15 μm and depth of 3 μm.

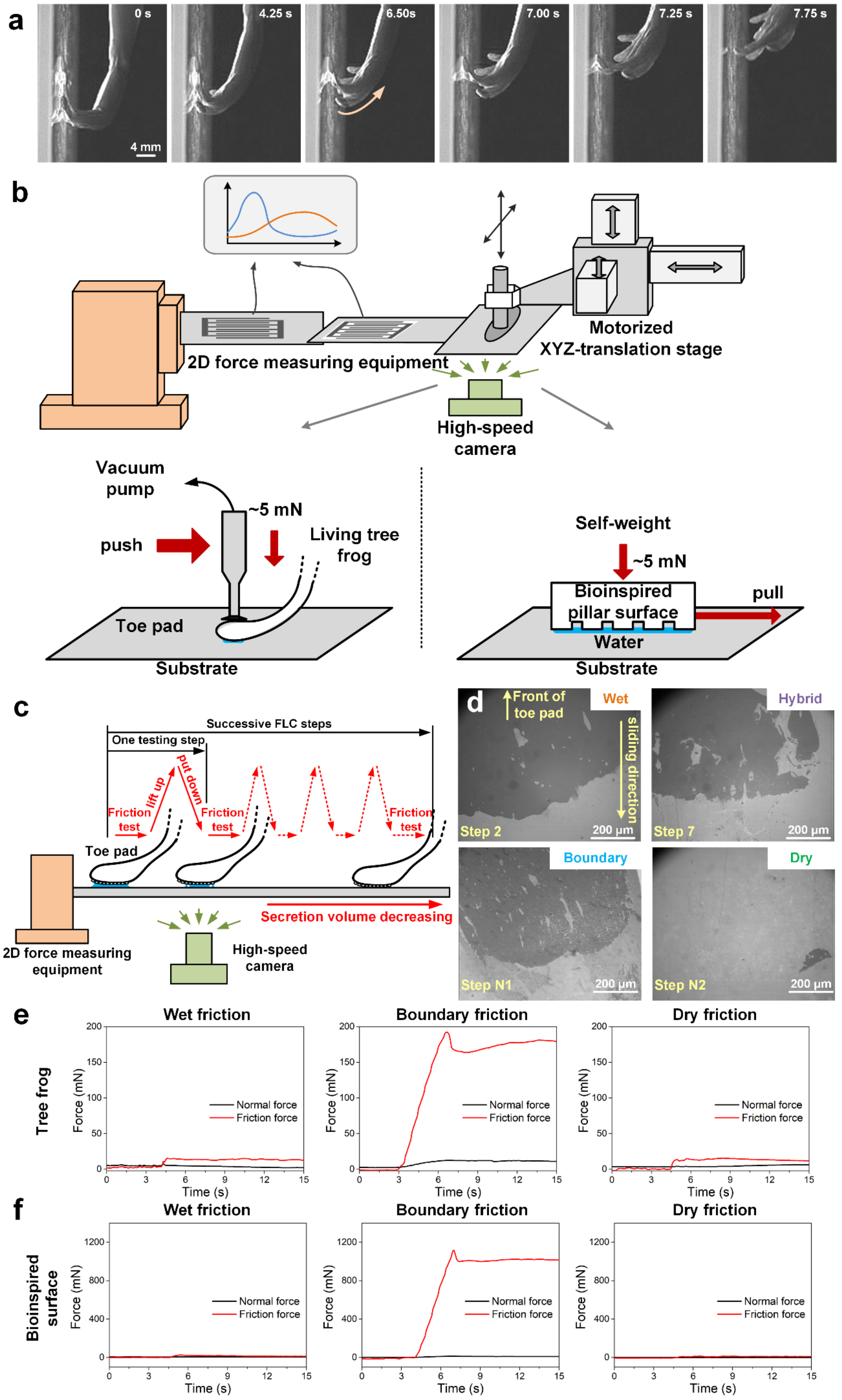
a
0 s
4.25 s
6.50s
7.00 s
7.25 s
7.75 s
4 mm
b
2D force measuring equipment
Motorized
XYZ-translation stage
High-speed
camera
Vacuum
pump
~5 mN
push
Living tree
frog
Toe pad
Substrate
Self-weight
~5 mN
Bioinspired
pillar surface
pull
Water
Substrate
c
Successive FLC steps
One testing step
Friction
test
lift up
put down
Toe pad
Secretion volume decreasing
2D force measuring
equipment
High-speed
camera
d
Front of
toe pad
Wet
sliding direction
Step 2
200 μm
Hybrid
Step 7
Boundary
Step N1
Dry
Step N2
e
Tree frog
Wet friction
Boundary friction
Dry friction
Force (mN)
Time (s)
Normal force
Friction force
f
Bioinspired
surface

Figure S3. Friction test for tree frog toe pad and bioinspired surfaces. (a) During tree frog climbing, its toe pads continuously detach and touch substrate in each gait. During detaching, the toe pad is gradually peeling off from substrate from one side (Movie S1). (b,c) Schematics of successive frog-like-crawling (FLC) steps test for tree frog toe pad and bioinspired pillar surfaces. Between each step, the toe pad is lifted up from a glass slide and put down on a new area on the glass slide. After each put down, the friction of toe pad is measured by a 2D force measuring equipment (minimum division value, 0.2 mN & max load, 10000 mN), with a normal force of ~5 mN. Secretion film state between the contact area is captured by a high-speed camera viewing from bottom. (d) Different secretion film states on toe pad in different steps. Secretion film between the toe pad and substrate can be recognized by the dark area in high-speed camera. (e,f) Typical boundary friction curve on tree frog's toe pad and bioinspired surface.

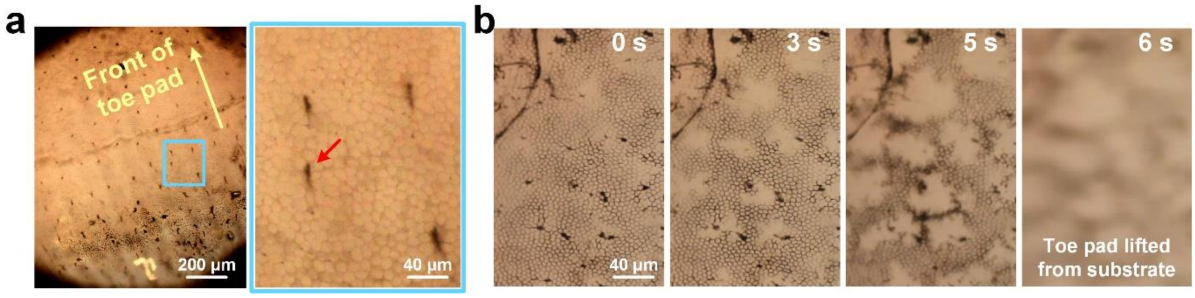

Figure S4. *In-situ* observation of secretion on tree frog's toe pad. (a) 2 µL Chinese ink (Carbon nanoparticle mixed liquid) is filled onto the toe pad. The glands denoted by dart dots are regularly distributed on the toe pad of tree frog. Red arrow denotes one gland surrounded by channels. (b) The glands simultaneously excrete secretion over the entire toe pad.

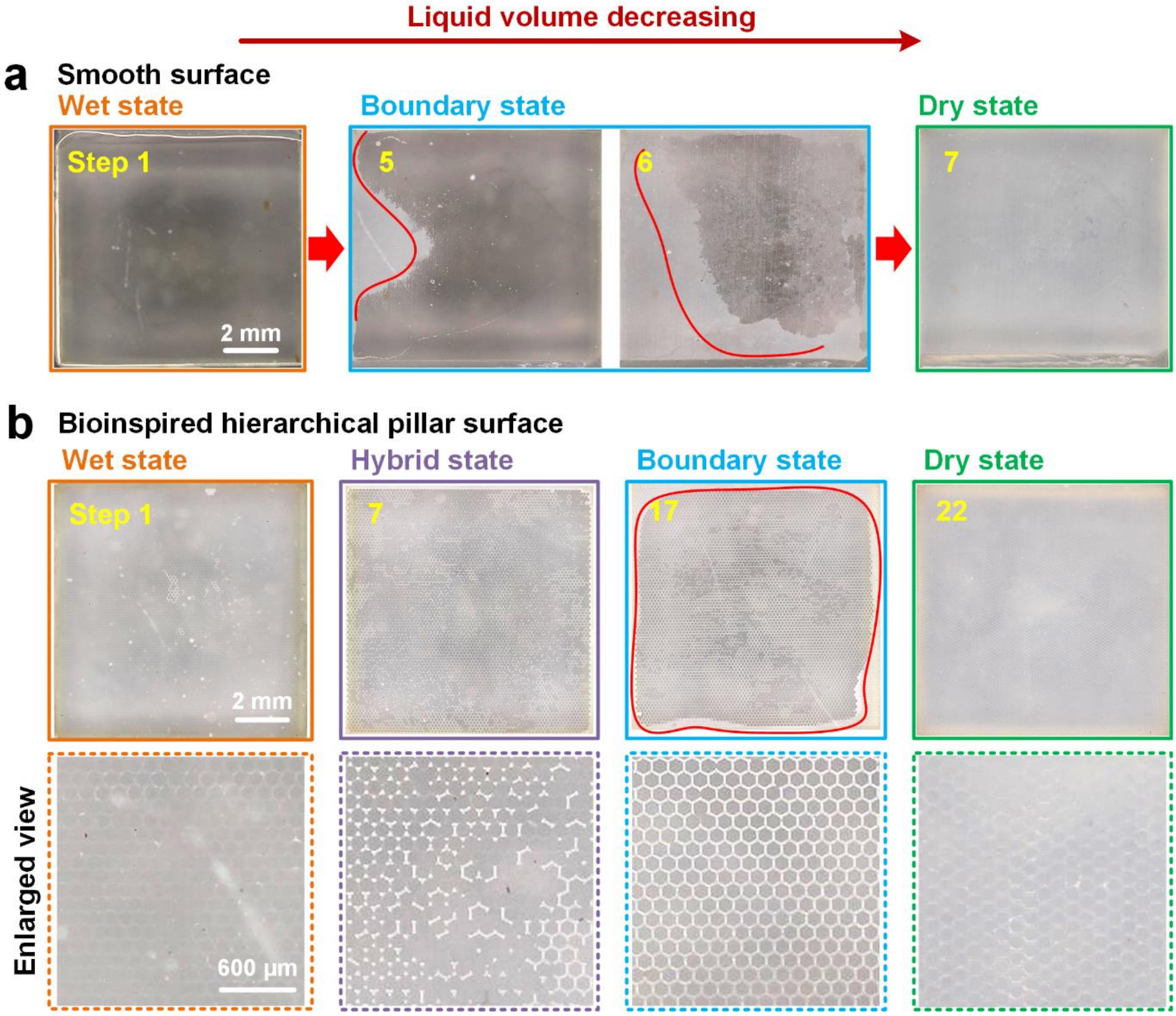

Figure S5. Liquid film state on smooth surface and bioinspired surface during successive FLC steps. (a) Liquid film state on smooth surface during successive FLC steps. Red curves outline the liquid film range. (b) Four types of liquid film state appear on bioinspired surface during successive FLC steps. Dashed Box is the enlarged view for different liquid film states.

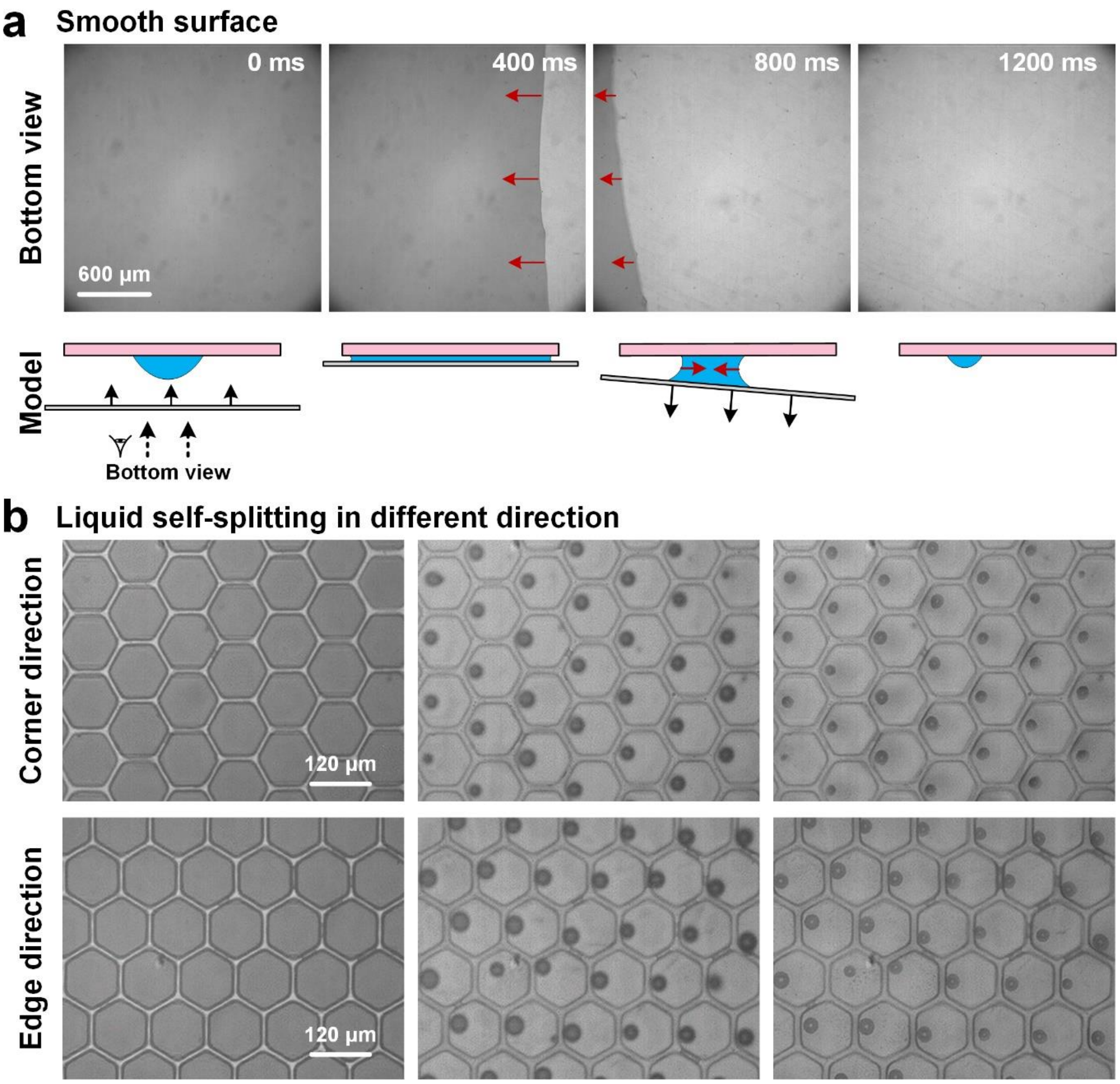

Figure S6. Characterization of liquid film during surfaces separating from substrate. (a) Liquid flows continuously on smooth surface during separation from substrate. (b) Liquid self-splitting happens in corner direction and edge direction.

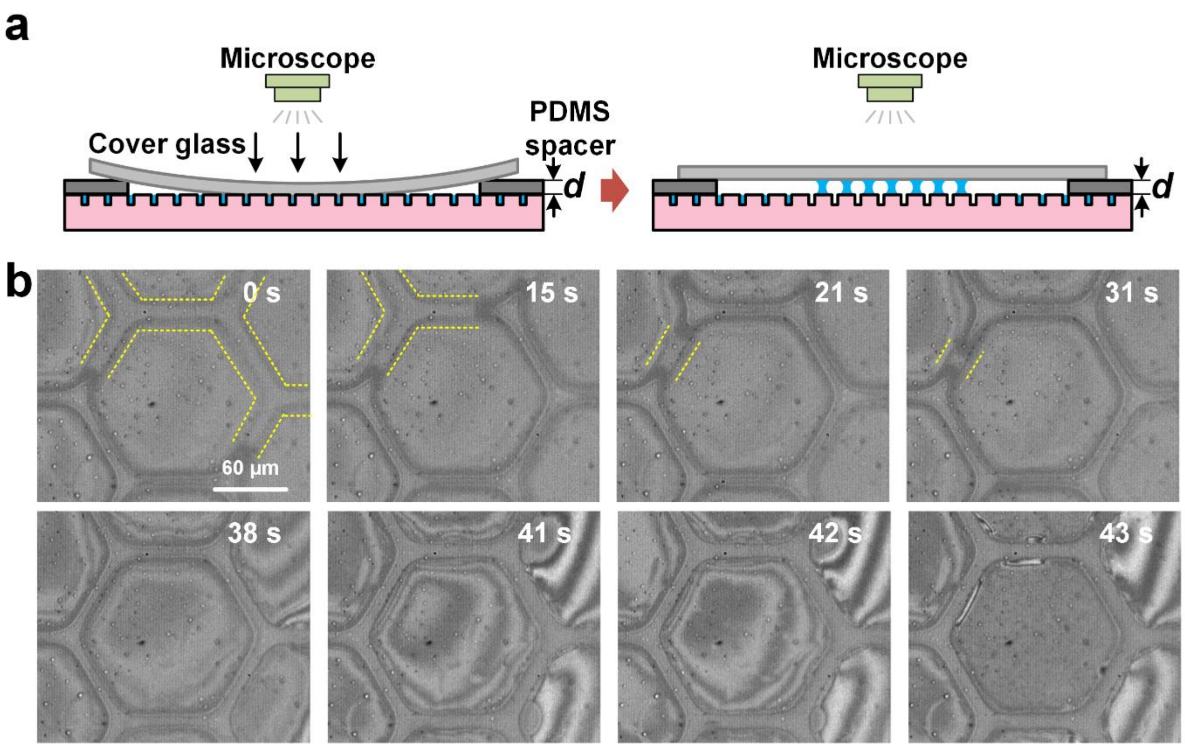

Figure S7. Mechanisms of liquid self-splitting on bioinspired surfaces. (a) To control the gap distance *d* to be higher than channel width *w*, two PDMS spacers are placed at two sides of bioinspired pillar surface. The PDMS spacers are achieved from spin-coating method and their thickness is controlled by adjusting spin-coating rotating speed. A cover glass crosses two spacers and forms a gap distance *d* over pillars. By pressing and releasing the center of cover glass, liquid self-splitting effect can be characterized through the microscope above. (b) With thin film interference (TFI) in the gap of pillar top and substrate, the change of gap distance *d* can be obtained during liquid film self-splitting by evaporation. Yellow dot lines denote the liquid distribution in channels. Liquid on pillar top is pinned by pillar edge during self-splitting through fluorescence observation.

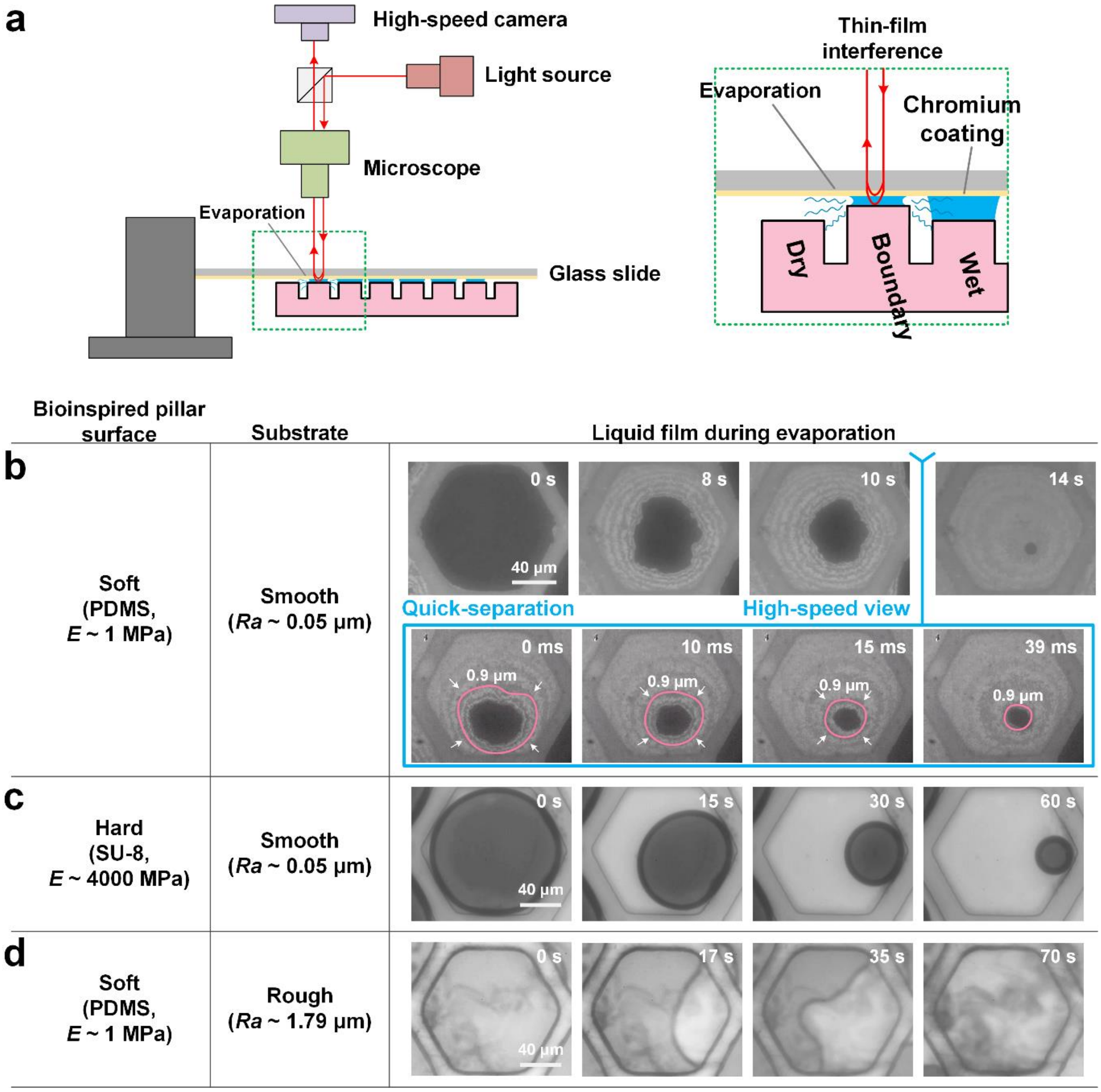

Figure S8. Characterization of pillar deformation induced by liquid bridge. (a) The tested surface is upside-down adhering to a glass slide by liquid. Thin-film interference forms between the interfaces. A high-speed camera and microscope are used to observe the interference fringes during liquid evaporation. (b) Nanometer-thick liquid bridge forms between soft micropillar (PDMS) and substrate. During liquid evaporating, interference fringes appear between the interfaces. At a certain moment, micropillar quickly separates from substrate and forms a relatively thicker liquid bridge. (c) Based on the evaporating process of liquid, thick liquid bridge forms between hard micropillar (SU-8) and smooth substrate, which leads to weak attachment. (d) Thick liquid bridge forms between soft micropillar and rough substrate with weak contact performance.

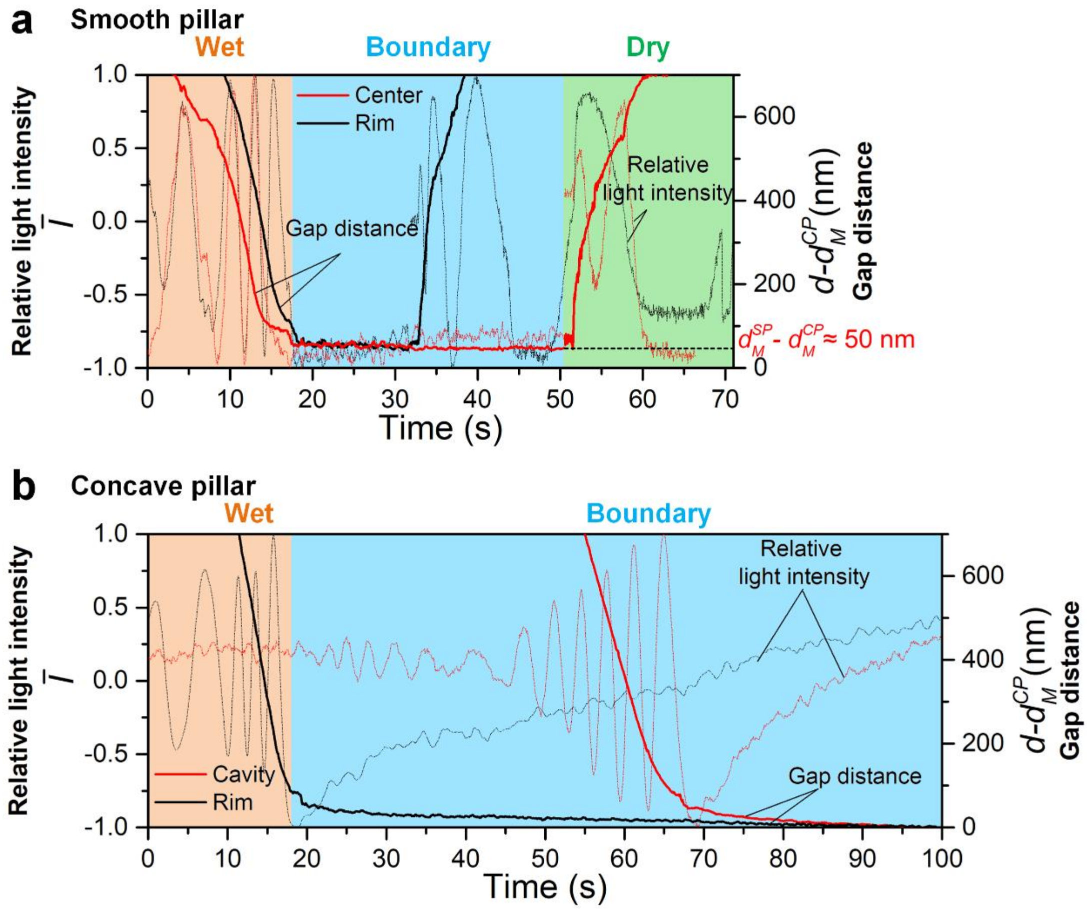

Figure S9. Characterization of gap distance between pillar and substrate with TFI. (a,b) By recording the relative light intensity on pillar $\bar{I}$ on smooth pillar and concave pillar, the gap distance at rim and center of these pillars are obtained. The d_M on concave pillar (d_M^{CP}) is measured ~50 nm thinner than on smooth pillar (d_M^{SP}).

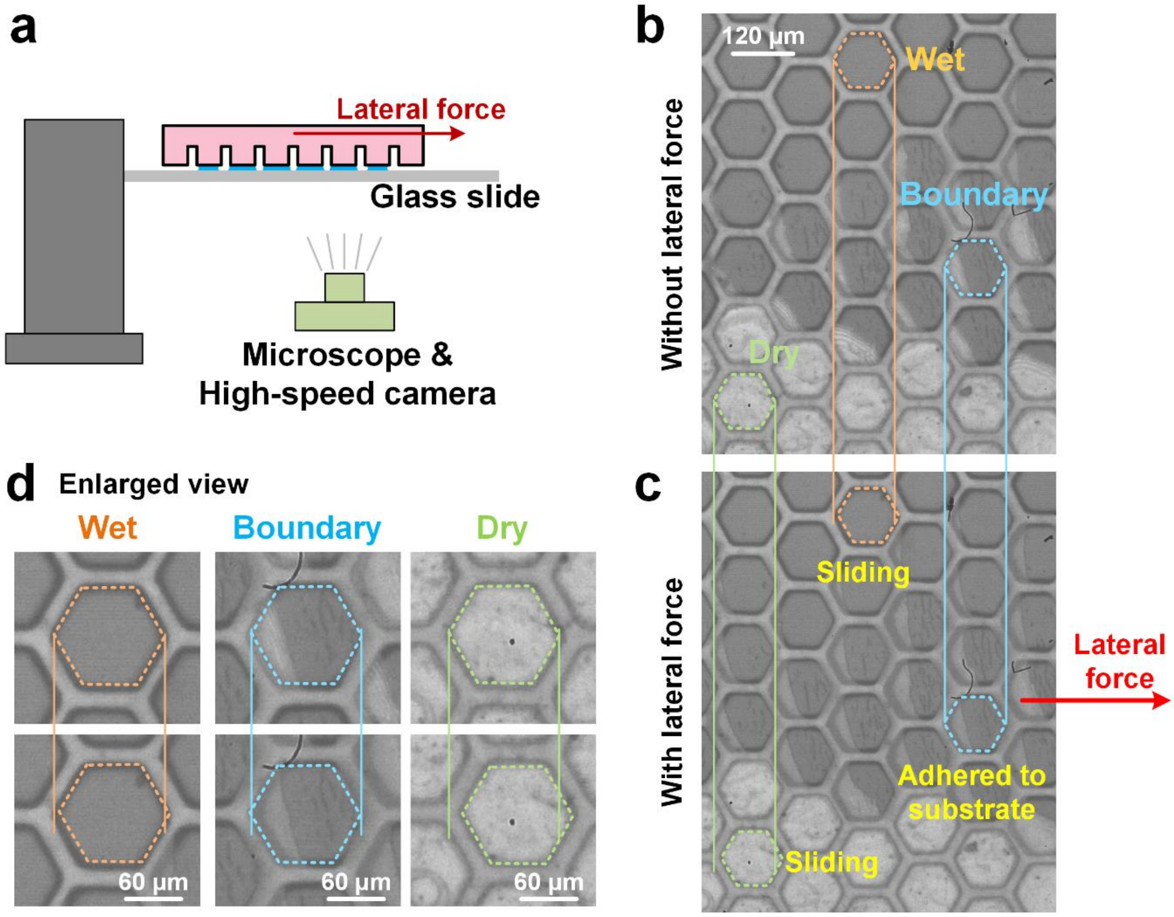

Figure S10. The deformation and friction of micropillars during bioinspired surface in hybrid state. (a) The setup of experiment. (b,c) Typical interfacial liquid film states in hybrid friction. The dark areas denote liquid film between pillar and substrate. When pillar is applied with lateral force, only pillars in boundary state demonstrate lateral deformation to generate friction, while pillars in wet and dry state exhibit negligible lateral deformation and provides no friction (Movie S7). (d) Enlarged view of pillar lateral deformation in different liquid film states.

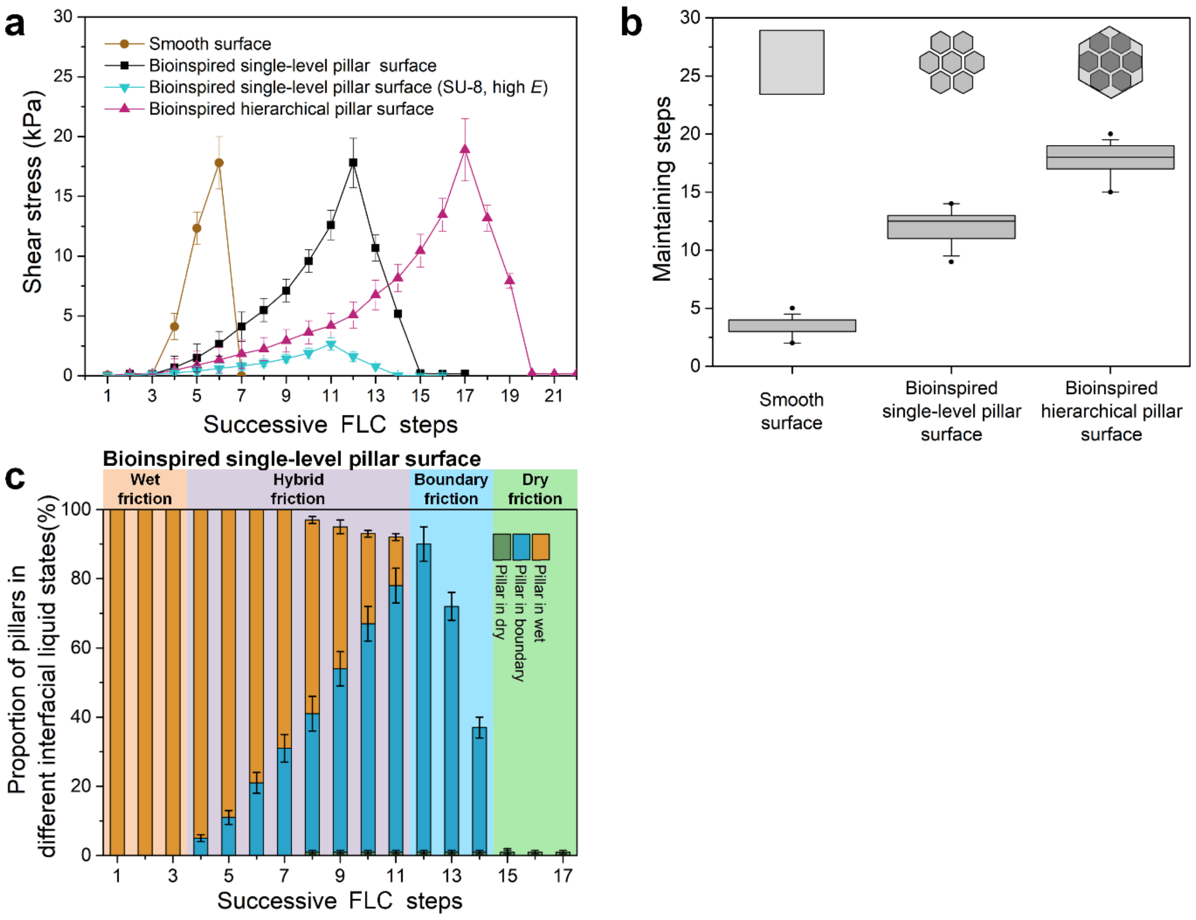

Figure S11. The friction characterization of bioinspired surfaces. (a) Friction in successive FLC steps for smooth surface, bioinspired single-level pillar surface and bioinspired hierarchical pillar surface. All these three types of surfaces are fabricated with soft PDMS (*E* ~1.8 MPa). Another bioinspired single-level pillar surface is fabricated with SU-8 photoresist (*E* ~2 GPa) to study the affection of material's elastic modulus *E*. Data are presented as mean ± SD, sample size n = 10. (b) The maintaining steps of hybrid and boundary friction for smooth surface, bioinspired single-level pillar surface and bioinspired hierarchical pillar surface on smooth substrate (sample size n = 10). It shows statistically significant difference in these three types of surfaces as determined by one-way ANOVA with $p < 0.05$. (c) On bioinspired surface, three types of liquid film state on pillars are observed including wet, boundary and dry states. The proportion of different liquid film states on pillars on bioinspired single-level pillar surface has been counted in each step (sample size n = 5). Hybrid friction forms when wet friction and boundary friction both appear on pillars.

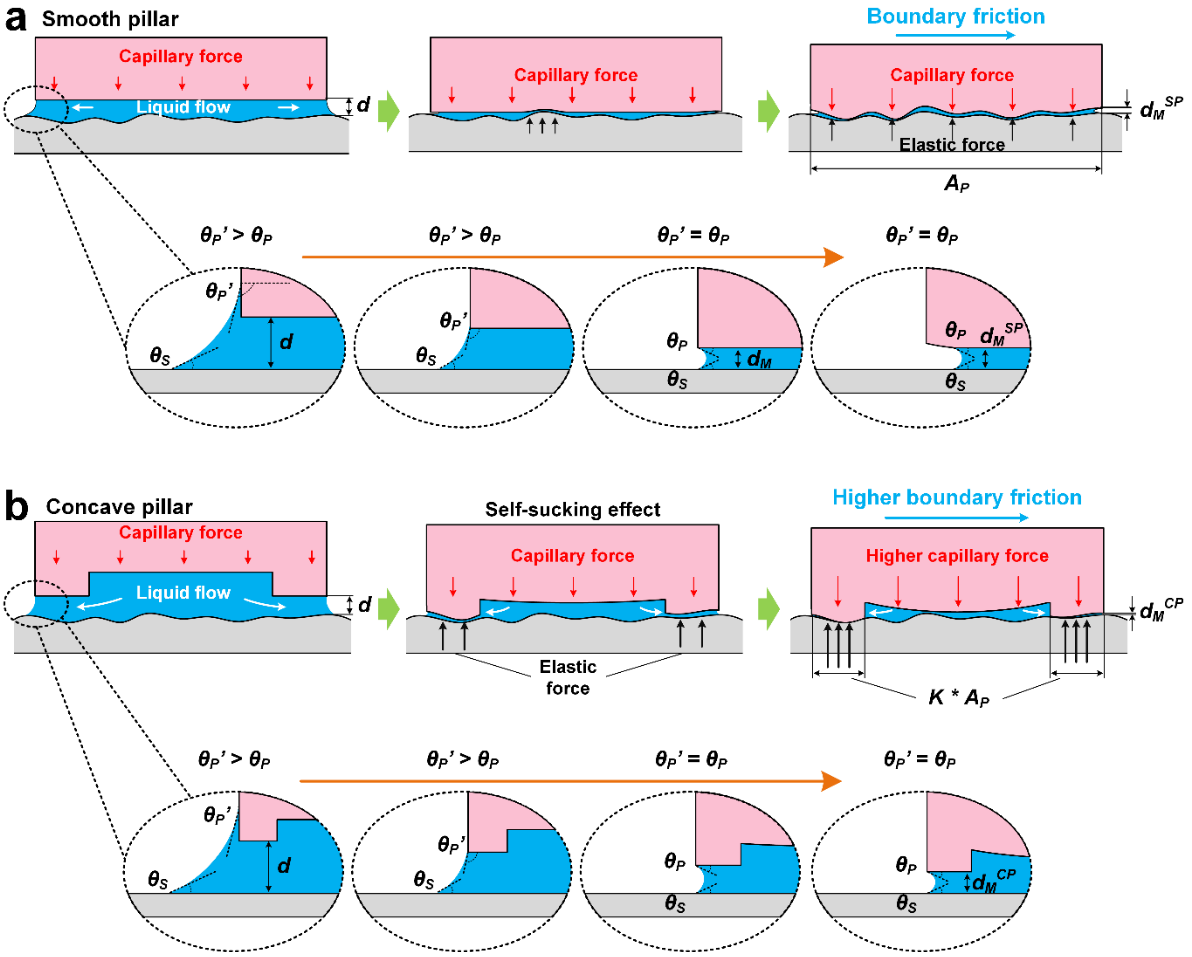

Figure S12. Theoretical analysis of rim self-sucking enhancing effect on concave pillar. With liquid strong capillary pressure, soft pillar is dragged to substrate and forms solid-solid contact with elastic deformation. (a) On smooth pillar with K = 1, the capillary bridge area and nominal contact area are the pillar area A_P. (b) On concave pillar during continuously evaporating, the pillar is gradually pulled to substrate, and solid-solid contact first forms at pillar rim (Left). Since the rim contact area $K * A_P$ is smaller than capillary force acting area A_P, solid-solid contact stress concentrates at rim area and leads to gap distance d lower with higher capillary pressure than on smooth pillar. With liquid keeps being sucked from cavity to rim area by capillarity to fill the evaporating vacancy, the cavity bottom is gradually stretched to substrate and forms the self-sucking effect (Middle). This self-sucking process further increases the capillary pressure and pressure concentration at rim area to form an even lower gap distance d_M^{CP}, which eventually leads to stronger boundary friction on concave pillar (Right). The theoretical analysis is presented in Section S3. During liquid evaporation, the apparent contact angle on pillar ϑ_P' varies with gap distance d, and eventually decreases to the intrinsic contact angle of pillar ϑ_P.

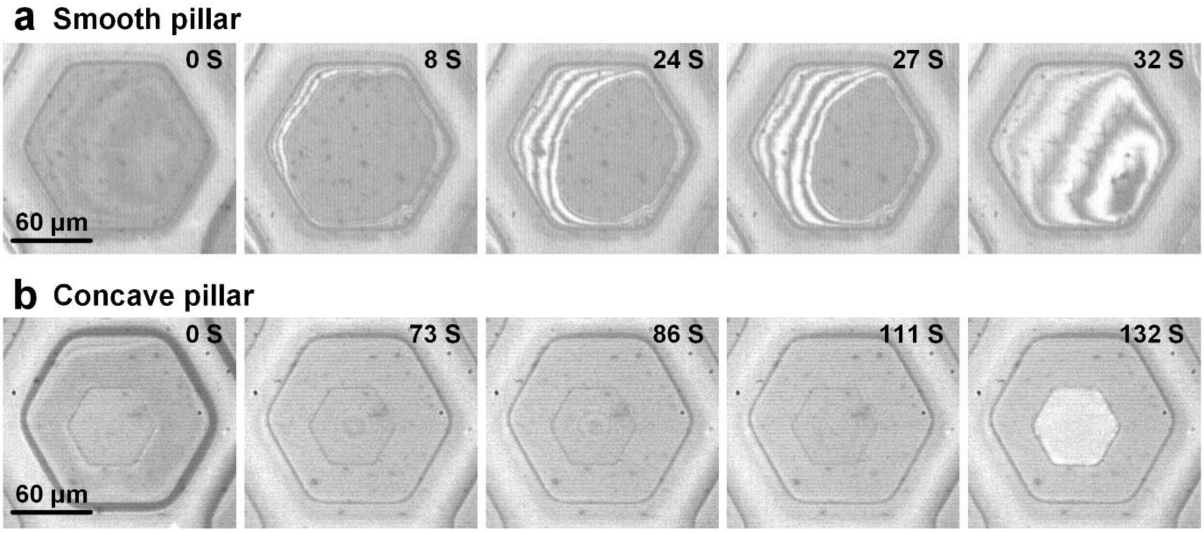

Figure S13. Comparison of boundary state duration on smooth pillar and concave pillar. The duration of boundary state on smooth pillar and concave pillar are recorded separately under the same condition to eliminate the mutual affection. Boundary state on (b) concave pillar with *K* ~0.65 maintains ~4 times longer than on (a) smooth pillar.

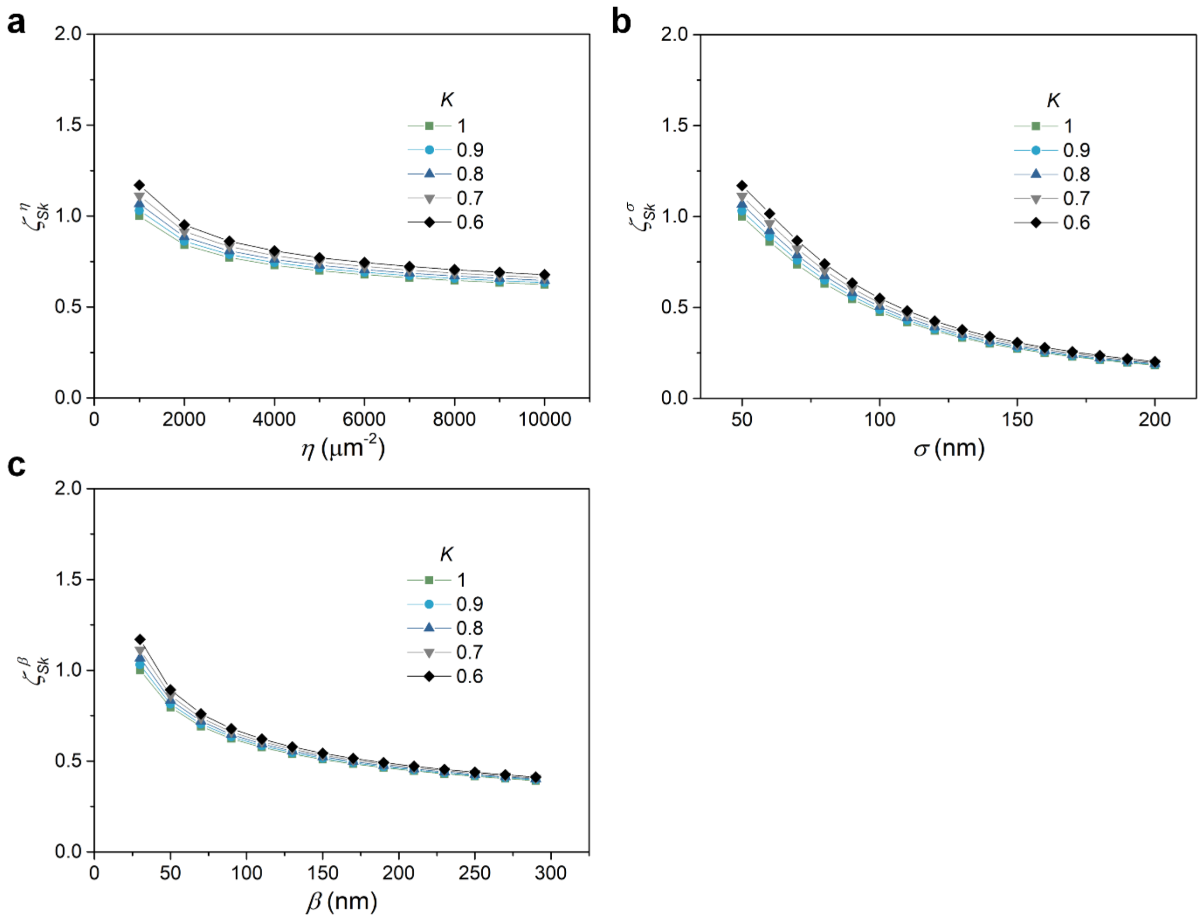

Figure S14. The influence of substrate roughness on boundary friction of pillar. With the increase of surface roughness, i.e. smaller η (a), σ (b) and β (c), weaker boundary friction is formed with smaller ζ_{Sk}^{η}, ζ_{Sk}^{σ} and ζ_{Sk}^{β}.

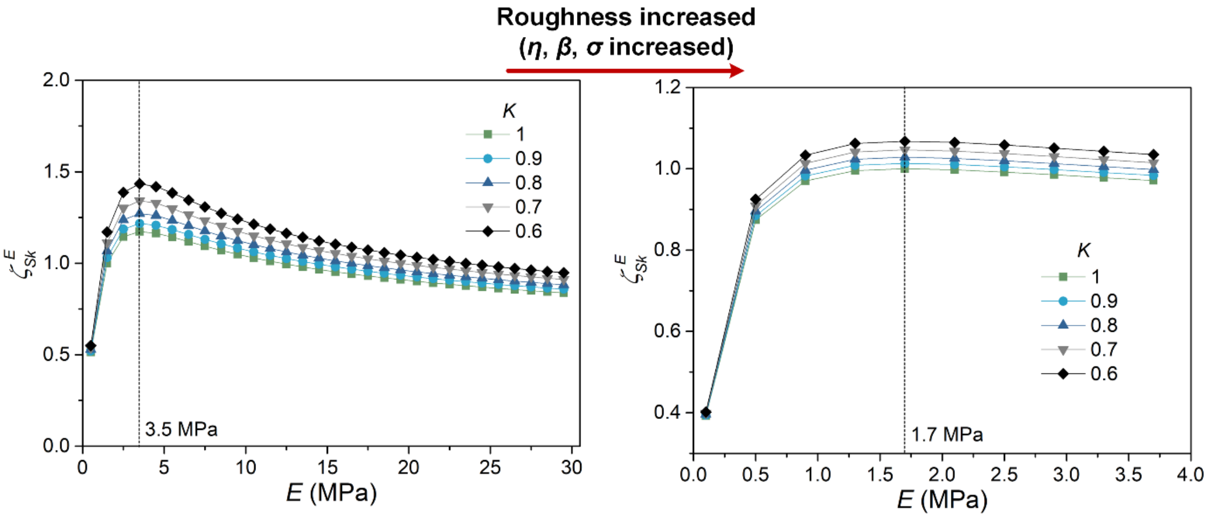

Figure S15. The influence of surface elastic modulus on boundary friction of pillar. For a certain substrate, the elastic modulus *E* of bioinspired surface exhibits an optimum value that can generate the highest boundary friction. With surface roughness increasing, the optimum *E* decreases which indicates softer bioinspired surface is suitable for rougher substrate.

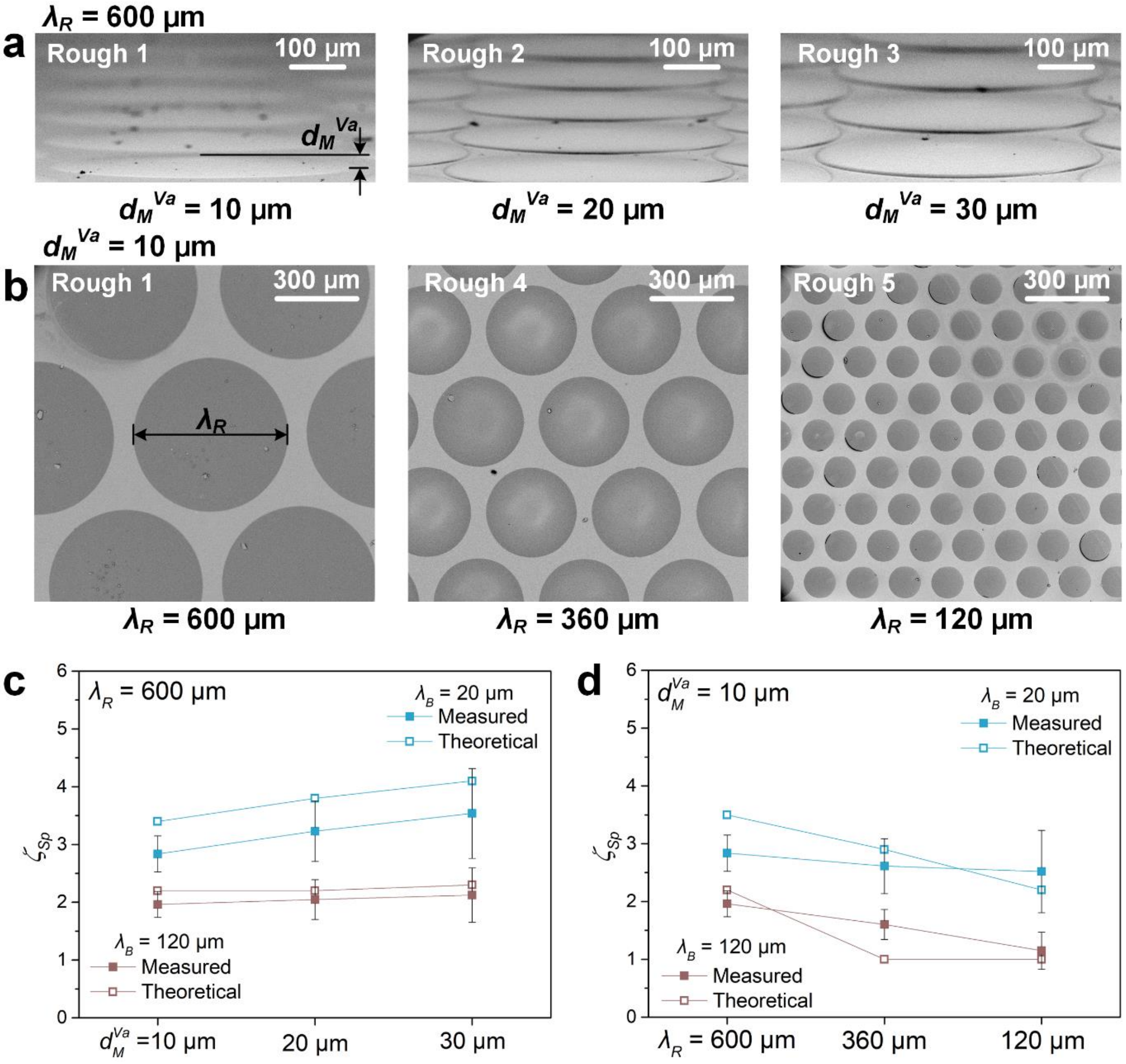

Figure S16. The self-splitting enhancing effect on different roughness substrates. (a,b) Tilting view and top view of 3D printed rough substrates (Rough 1-5), which are constructed with hexagonally arrayed spherical crowns. The height and diameter of spherical crown represent substrate roughness properties, including gap distance at bump valley $d_M{}^{Va}$ and rough wavelength λ_R, respectively. Rough 1 to 3 are fabricated with an increasing $d_M{}^{Va}$ and Rough 1,4,5 are fabricated with a decreasing λ_R (Table S3), which both have increasing roughness. (c,d) The comparison of ζ_{Sp} between measured friction and theoretical value from Equation S27.

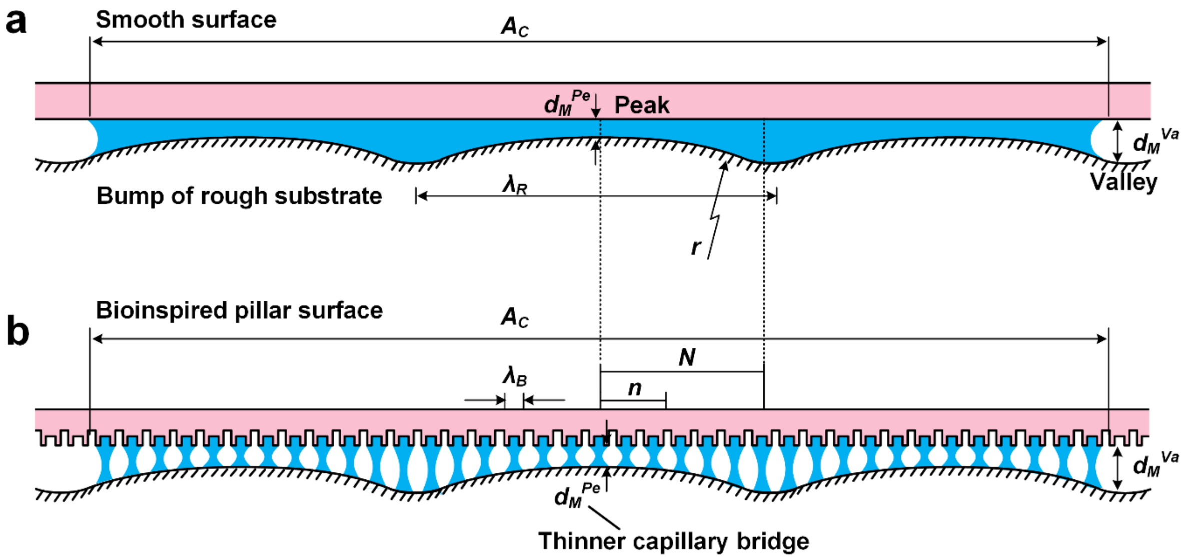

Figure S17. Theoretical analysis about self-splitting enhancing effect on bioinspired pillar surface. (a) On smooth surface, liquid gathers around the spherical rough bump and forms a thick liquid film at bump valley with large minimum gap distance d_M^{Va}. (b) On bioinspired pillar surface, pillars could have much smaller minimum gap distance at bump peak d_M^{Pe} and form strong capillary force. λ_R is the wavelength of roughness on substrate, here it is the diameter of the spherical bump section. λ_B is the diameter of pillar. A_C is the capillary bridge area on smooth surface and bioinspired pillar surface, respectively. r is the radius of spherical rough bump.

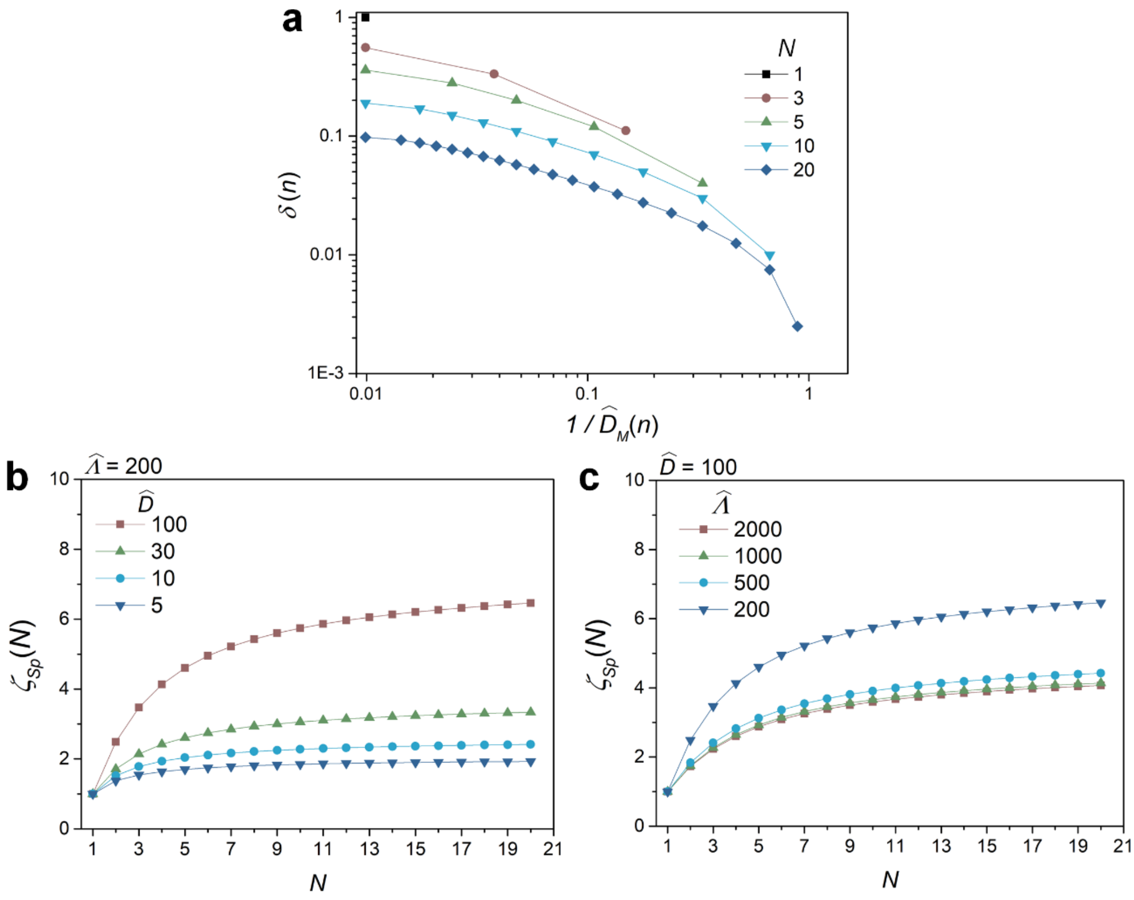

Figure S18. The self-splitting enhancing effect on bioinspired pillar surface. (a) With the number of pillars N increasing, thinner capillary bridges with larger $1/\widehat{D}_M(n)$ appears, thus stronger boundary friction forms. Here, $\hat{\Lambda} = 200$, $\widehat{D} = 100$. (b,c) The self-splitting enhancing coefficient $\zeta_{Sp}(N)$ increases with number of pillars N. It increases with the growing of normalized minimum gap distance at bump valley $\widehat{D}$ and the decreasing of normalized substrate roughness wavelength $\hat{\Lambda}$.

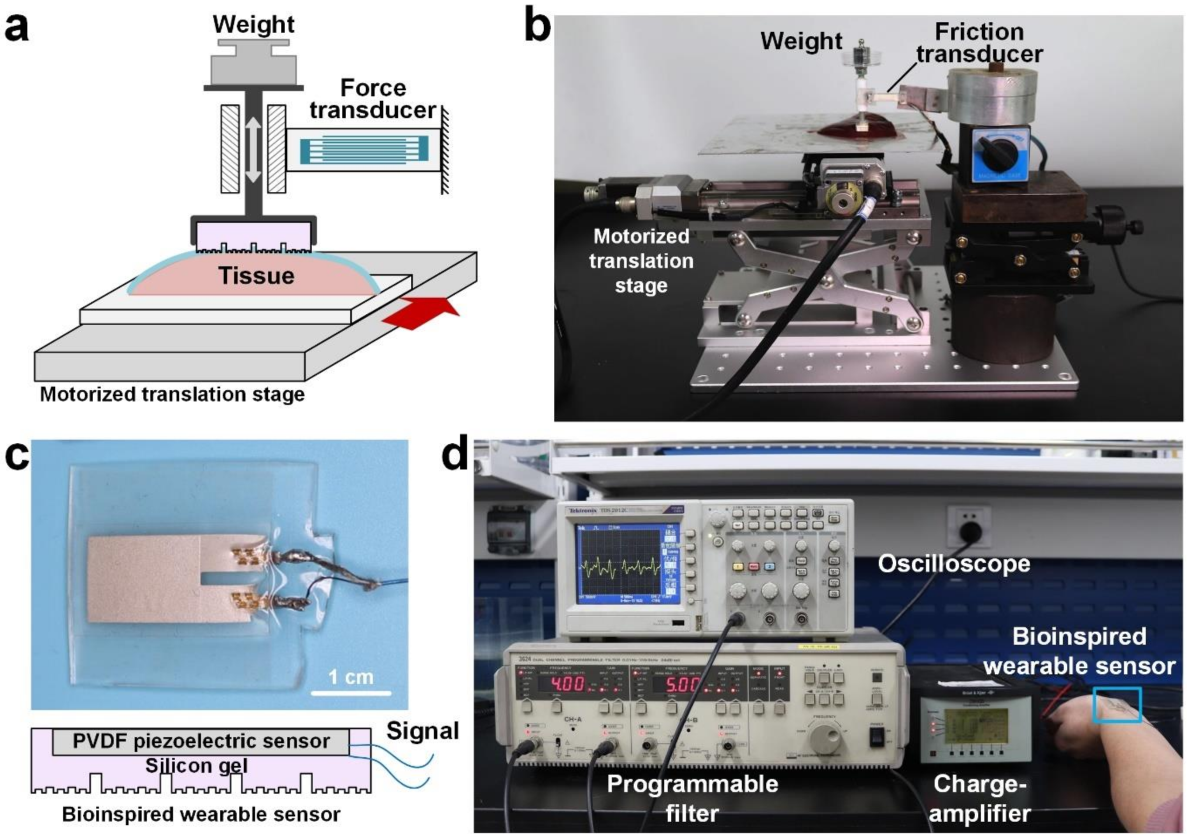

Figure S19. Test equipment setup for bioinspired surgical graspers and bioinspired wearable sensors. (a,b) The friction of bioinspired surface and 1 mm sharp teeth surface has been tested on fresh pig liver with different weights. (c) The bioinspired wearable sensor embedded with a PVDF piezoelectric sensor is replicated from a bioinspired surface model by silica gel. (d) The electrical signals generated by pulse vibration are processed by a charge amplifier and a low-pass filter, then displayed by an oscilloscope. During test, different volumes of water are added to skin by a pipette to simulate different states of sweat.

Table S1. Structural parameters of different bioinspired surfaces (area 1 cm × 1 cm).

Type		Pillar/cavity diameter	Pillar height/cavity depth	Channel width
		a (μm)	*h* (μm)	*w* (μm)
Bioinspired single-level pillar		120	30	20
Bioinspired hierarchical pillar	First level	120	30	20
	Second level	20	10	3
Bioinspired hierarchical concave pillar	First level	120	30	20
	Second level	20	10	3
	Cavity	6, 12, 15	3	

Table S2. Numerical solution with different ratio of rim area to pillar area K. d_M is the minimum gad distance, $M(d)$ real contact area ratio, $\zeta_{Sk}(K)$ boundary friction enhancing coefficient of self-sucking, and ζ_{Sk}^{t} boundary friction duration enhancing coefficient of self-sucking.

K	1	0.9	0.8	0.7	0.6	0.5	0.4
d_M (nm)	223	213	202	190	175	157	135
$M(d)$	0.27	0.31	0.37	0.44	0.54	0.70	0.96
$\zeta_{Sk}(K)$	1	1.03	1.07	1.12	1.18	1.27	1.40
ζ_{Sk}^{t}	1	1.77	2.70	3.89	5.64	9.02	28.32

Table S3. Structural parameters of different rough substrates (area 2 cm × 2 cm).

	Minimum gap distance at bump valley d_M^{Va} (μm)	Substrate roughness wavelength λ_R (μm)
Rough 1	10	600
Rough 2	20	600
Rough 3	30	600
Rough 4	10	360
Rough 5	10	120

Movie S1. Tree frog toe pad peeling off from substrate during a crawling step (400 fps).

Movie S2. The flow of liquid on smooth surface and bioinspired surface during separation from substrate. A continuous liquid film appears on smooth surface while self-split liquid films appear on bioinspired surface.

Movie S3. Liquid self-splitting in successive FLC steps.

Movie S4. Liquid self-splitting during evaporating.

Movie S5. Different TFI on smooth pillar and concave pillar induce by liquid strong capillarity.

Movie S6. The quick-separation on micropillar during liquid evaporation. Since liquid volume remains almost unchanged during quick-separation, liquid film thickness before quick-separation can be obtained by comparing the area changing before and after quick-separation.

Movie S7. Different lateral deformation on pillars in wet, boundary and dry states. Boundary state pillar with nanometer-thick liquid film forms the largest lateral deformation, i.e the strongest friction.

Movie S8. Comparison of boundary friction duration on smooth pillar and concave pillar. The duration of boundary state on concave pillar is ~4 times higher than on smooth pillar.

References

[1] J. Luo, S. Wen, P. Huang, *Wear* **1996**, 194, 107.

[2] P.-G. de Gennes, F. Brochard-Wyart, D. Quere, *Capillarity and Wetting Phenomena: Bubbles, Pearls, Waves*, Springer-Verlag, New York, 2004.

[3] J. Greenwood, J. P. Williamson, *Proc. R. Soc. Lond. A* **1966**, 295, 300.

[4] B. Lorenz, Y. R. Oh, S. K. Nam, S. H. Jeon, B. N. J. Persson, *J. Chem. Phys.* **2015**, 142, 194701.

[5] B. Lorenz, B. A. Krick, N. Rodriguez, W. G. Sawyer, P. Mangiagalli, B. N. J. Persson, *J. Phys.: Condens. Matter* **2013**, 25, 445013.

[6] B. N. J. Persson, *J. Chem. Phys.* **2001**, 115, 3840.
